# Diagnosis of subarachnoid haemorrhage: Systematic evaluation of CT head diagnostic accuracy and comparison with the 2022 NICE guidelines

**DOI:** 10.1016/j.bas.2025.104200

**Published:** 2025-02-04

**Authors:** Conor S. Gillespie, John Gerrard Hanrahan, Roxana Mahdiyar, Keng Siang Lee, Mohammad Ashraf, Ali M. Alam, Justyna O. Ekert, Orla Mantle, Simon C. Williams, Jonathan P. Funnell, Nihal Gurusinghe, Raghu Vindlacheruvu, Peter C. Whitfield, Rikin A. Trivedi, Adel Helmy, Peter J. Hutchinson

**Affiliations:** a Department of Clinical Neurosciences, University of Cambridge, Cambridge, UK; b Department of Neurosurgery, Addenbrooke's Hospital, Cambridge, UK; c Department of Neurosurgery, The Walton Centre NHS Foundation Trust, Liverpool, UK; d Department of Neurosurgery, National Hospital for Neurology and Neurosurgery, London, UK; e Wellcome/EPSRC Centre for Interventional and Surgical Sciences (WEISS), London, UK; f School of Medicine, University of Lancaster, Lancaster, UK; g Department of Basic and Clinical Neurosciences, Maurice Wohl Clinical Neuroscience Institute, Institute of Psychiatry, Psychology and Neuroscience, King's College London, London, UK; h Department of Neurosurgery, Institute of Neurological Sciences, Queen Elizabeth University Hospital, Glasgow, UK; i Institute of Infection, Veterinary, and Ecological Science, University of Liverpool, UK; j Department of Neurosurgery, The Royal London Hospital, London, UK; k Department of Neurosurgery, Lancashire Teaching Hospitals NHS Foundation Trust, Preston, UK; l Department of Neurosurgery, Barking, Havering and Redbridge, University Hospitals NHS Trust, UK; m South West Neurosurgery Centre, University Hospitals Plymouth NHS Trust, Plymouth, UK

**Keywords:** Subarachnoid haemorrhage, Lumbar puncture, LP, CT, Computed tomography, 6 h

## Abstract

**Introduction:**

Aneurysmal subarachnoid haemorrhage has a high incidence, and morbidity. It has been suggested that a negative non-contrast CT head can rule out SAH if performed within 6 h of symptom onset.

**Research question:**

What is the sensitivity of CT head at ruling out SAH stratified by time-point, and what is the potential impact of omitting Lumbar Puncture (LP) from the diagnostic pathway?

**Material and methods:**

Systematic review and meta-analysis (PROSPEROID CRD42022379929). Three databases were searched, and articles published between January 2000–May 2022 included (Search date 27^th^ November 2022). Primary objective was diagnostic accuracy of CT scans for detecting SAH at <6 h from symptom onset, including reported sensitivity, and specificity values.

**Results:**

63 articles were included (38,237 patients, 7673 with SAH). Pooled CT head sensitivity was 0.94 for excluding SAH (22 studies, 95% Confidence Interval [CI] 0.90–0.97). At <6 h, CT head sensitivity was 0.995 (6 studies, 95% CI 0.941–1.000). Most studies (57.1%, n = 36/63) were classified as high risk of bias. If LP was removed from the diagnostic pathway in the UK, assuming an incidence of 4800 SAH per-year, 336 SAH would be missed per-year, 24 per-year if LP was removed for negative CT < 6 h (95% CI 0–278) and 58 per-year if mean sensitivity is used (95% CI 0–240).

**Discussion and conclusion:**

CT head appears to be highly sensitive at excluding SAH <6 h from symptom onset. High quality, prospective data is required to further established the utility of early (<6 h) negative CT head. We recommend that if there is strong clinical suspicion of SAH, yet CT head is reported negative <6 h of symptom onset, that a LP be performed.

## Highlights

• Systematic Review and Meta-Analysis of over 60 studies from 3 separate databases.
• Data based on 7673 patients with multiple sensitivity analysis performed.
• Provides precise sensitivity values for CT head in diagnosing SAH at different time-points from symptom onset.
• Only six studies contained pooled data on primary outcome (sensitivity <6 h).
• Our estimation of missed cases assumes all cases of SAH have a CT < 6 h, which does not reflect clinical practice.

## 1 Introduction

Headache accounts for a significant proportion of neurological emergencies and approximately 100,000 hospital admissions in the UK(Chapman et al., 2009), (Burch et al., 2021; Southwell et al., 2021). Whilst less than 2% of these admissions arise as a result of serious intracranial pathology(Southwell et al., 2021), life-threatening conditions such as subarachnoid haemorrhage (SAH) must be excluded. SAH affects 2.3–21.5 in 100,000 people every year(de Rooij et al., 2007) and has a high morbidity- (Sandvei et al., 2011) therefore clinicians must consider and rule it out.

SAH is mainly diagnosed by a non-contrast CT head scan(Lansley et al., 2016) which is sensitive in detecting SAH (Perry et al., 2011; Backes et al., 2012). However, when SAH is suspected but a CT head does not reveal blood, a diagnostic lumbar puncture was traditionally recommended(NICE, 2022). In recent years, the diagnostic utility of LP has been questioned, and the sensitivity of modern CT scanners has increased. Evidence suggests that scan sensitivity increases the sooner patients are scanned(Vincent et al., 2022; Matthew et al., 2022), with a higher sensitivity seen in those scanned within 24 h of symptoms onset (Vincent et al., 2022; Dubosh et al., 2016).

In November 2022, the National Institute for Health and Care Excellence (NICE) published guidelines recommending that if a CT head was performed within 6 h of symptom onset and reported by a radiologist as showing no evidence of SAH, then it was excluded and to not routinely offer a lumbar puncture (NICE, 2022). However, many clinicians still believe LP to be a crucial diagnostic tool in diagnosing SAH (Martin et al., 2015). The current literature has been synthesised, but has never been fully explored in its content, or quality, which is vital to ensuring best practice is followed (Riley et al., 2019).

The primary objective of the study was as follows: to identify the diagnostic utility of CT head stratified by time points, at diagnosing SAH. Secondary objectives were to identify the diagnostic utility of LP, and to critically evaluate the quality of available literature to assess the recent NICE recommendations.

## 2 Methods and analysis

### 2.1 Search strategy and selection criteria

We conducted a systematic review and meta-analysis according to the Preferred Reporting Items for Systematic Reviews and Meta-Analyses (PRISMA) guidelines (Page et al., 2021). The review was registered in PROSPERO (CRD42022379929). Following registration, it was decided to exclude studies published before the year 2000.

We searched Medline, Embase, and the Cochrane database of systematic reviews for full-text articles published in English or with a clear translation (Nussbaumer-Streit et al., 2020), between the publication date 1^st^ January 2000 and 27^th^ November 2022. The date of last search was 27th November 2022. Search terms used a combination of the words ‘subarachnoid haemorrhage’ and ‘lumbar puncture’ (Full search strategy available in Supplementary Tables 1–4). The Population, Intervention, Comparator, Outcome, Study Design (PICOS) criteria were used, and are shown in Supplementary Table 5. We included studies of adults (≥18 years) presenting with any symptoms suggestive of, or diagnosed with SAH that reported the number diagnosed with a non-contrast CT head, and/or Lumbar Puncture. We excluded conference abstracts, case reports, and studies published before the year 2000 (as these were felt to almost certainly be out of date due to the advent of new generation CT scanners) (Boesiger et al., 2005; Gee et al., 2012). We excluded studies with selective populations (i.e ones that did not follow the traditional diagnostic pathway of CT head followed by LP, such as investigating with LP first then CT), non-clinical studies, and studies where no diagnostic information as available. Two reviewers independently screened titles, abstracts and full-text to include articles. If reviewers failed to reach consensus, a third author made a final determination. Summary estimates level data was sought.

### 2.2 Data extraction

Data extraction was completed in duplicate by at least two authors per paper and included: year published, journal of publication, study design (RCT, prospective or observational), country, interpreter of CT (Radiologist, Neuroradiologist, both, mixed) and author subspeciality.

Numerical data were extracted from each study: total population; total population with SAH diagnosed; number diagnosed by CT; number diagnosed by LP; true positive, true negative, false positive, and false negative for CT head (any stage), CT head <6 h, CT head <12 h, CT head <24 h, LP); number of cases missed if LP was removed from each study in the diagnostic pathway (n, %); and number diagnosed with CTA, MRA or other investigations. Definitions of datapoints are included in Appendix A. All reported reference standards were considered acceptable for inclusion.

### 2.3 Quality assessment

Retrospective studies were classified according to the Newcastle Ottawa Scale (Stang, 2010), and RCTs were assessed according to the Cochrane Risk of Bias 2.0 tool (Sterne et al., 2019).

### 2.4 Statistical analysis

For the meta-analysis, we used random effects models of variables and end-points. Bivariate summary receiver operating characteristic (SROC) curves and point estimates of sensitivity and specificity (principal diagnostic accuracy measures per-patient) were generated using bivariate random-effects meta-analysis models in the approach by Plana et al. (2022). We evaluated the performance of CT head at different time points through crossover diagrams and diagnostic odds ratios (DOR). We summarised findings using pooled forest plots for sensitivity and specificity, and ROC plane plots. Bivariable analysis were presented when possible. When studies were nondivergent, univariable analysis and ROC plane plots were used (Lee et al., 2022). For comparison, we also included the combined mean of calculated sensitivity values.

Sensitivity analyses where performed for studies at high risk of bias, authorship group, and studies published by ED departments. To estimate the effect of omitting LP from the diagnostic pathway, we used the pooled sensitivity values, and an established incidence of 4800 SAH diagnosed per year, to approximate the number of patients that would be missed (RCS(Eng), 2006).

Data analysis of descriptive statistics was performed using SPSS (Version 27; IBM; Armonk; NY; USA). R statistics (Rstudio Version 4.0.1) was used to perform meta-analysis, using a web application for meta-analysis of diagnostic test accuracy data (MetaDiSc 2.0, http://www.metadisc.es/) (Plana et al., 2022).

### 2.5 Patient and public involvement

As this was a systematic review and meta-analysis of published studies, patients/the public were not involved in the concept or design of this study.

## 3 Results

**Fig. 1:**
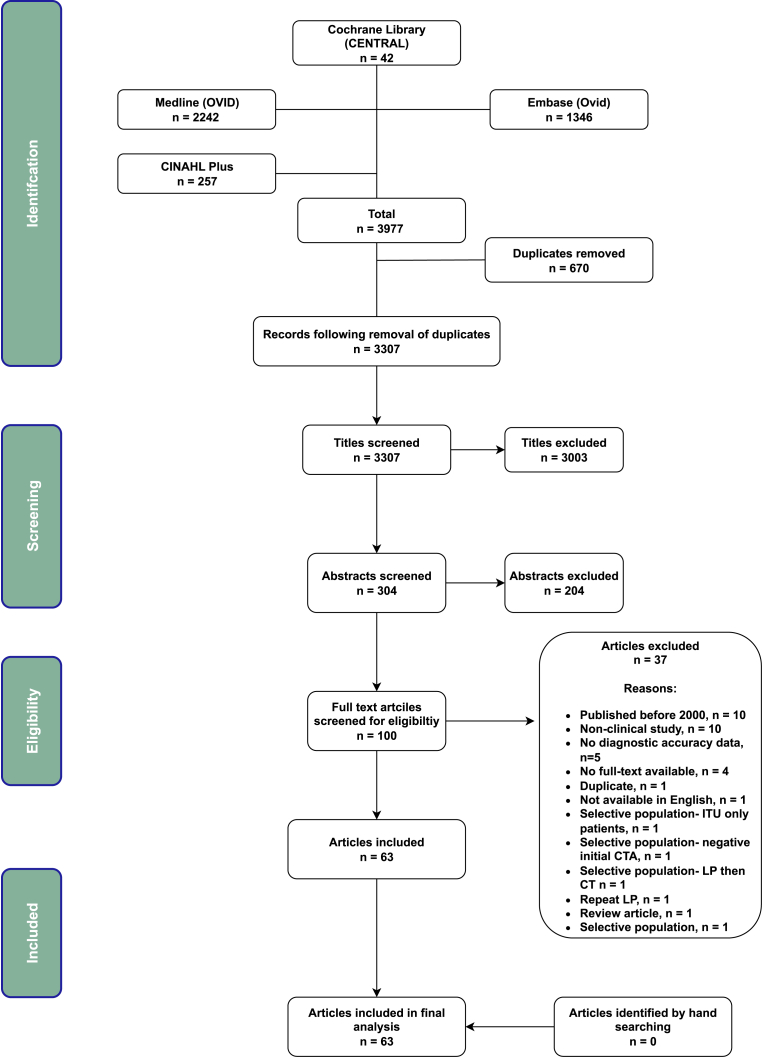
PRISMA Flow diagram, of study selection for inclusion in this review and meta-analysis.

63 out of 304 possible studies were included in the final analysis (Fig. 1). In total, 50 (79.4%) of studies were retrospective, and 13 (20.6%) studies were prospective.

The baseline characteristics of included studies are shown in detail in Table 1. The median number of patients included per study was 255 (IQR 123–644, range 12–4407), with the median number of diagnosed SAH per study 15 (IQR 6–134, range 0–1657). Fifteen (23.8%) studies were multi-centre. Twenty-three (36.5%) studies examined CT diagnostic accuracy, and 19 (30.2%) LP accuracy. 52 (82.5%) studies specified a reference standard, most commonly LP (46.0%, N = 29). For CT reporting 27% (N = 17) used a radiologist only, 18% (N = 11) a neuroradiologist, 18% (N = 11) either a radiologist or neuroradiologist, and 33% (N = 21) did not state the reporting physician.

**Table 1:** Baseline characteristics.

**Characteristic**	**Value**
Total studies included	63
Median patient number (IQR)	255 (12–4407)
Median confirmed SAH (IQR)	15 (6–134)
Published 2010–2022 (%)	47 (74.6)
**Journal**	**Frequency (%)**
Emergency Medicine Journal	4 (6.3)
British Journal of Neurosurgery	4 (6.3)
Stroke	3 (4.8)
Other	52 (82.5)
**Country**	**Frequency (%)**
United Kingdom	21 (33.3)
USA	11 (17.5)
Canada	8 (12.7)
Netherlands	6 (9.5)
Other	17 (27.0)
**Diagnostic accuracy**	**Frequency (%**)
Studies including CT data (any time point)	22 (34.9)
Studies including CT data (<24 h)	3 (4.8)
Studies including CT data (<12hrs)	3 (4.8)
Studies including CT data (<6 h)	6 (9.5)
Studies including LP accuracy data	34 (54.0)

**Fig. 2:**
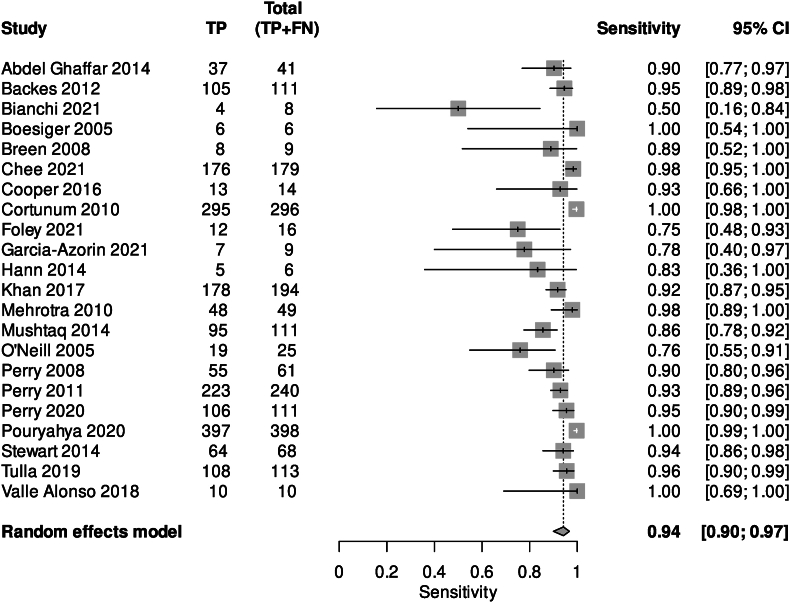
Sensitivity forest plot for CT head at any time point.

The accuracy of CT at any time point is shown in Table 2 and Fig. 2 (Abdel Ghaffar et al., 2014; Bianchi et al., 2021; Breen et al., 2008; Chee et al., 2021; Cooper et al., 2016; Cortnum et al., 2010; Foley, 2021; Garcia-Azorin et al., 2021; Hann et al., 2015; Khan et al., 2017; Mehrotra et al., 2010; Mushtaq et al., 2014; O'Neill et al., 2005; Pouryahya et al., 2020; Stewart et al., 2014; Tulla et al., 2019). 33 studies (52.4%) reported CT diagnostic accuracy at any time point (or did not specify). Among the 22 studies that could be pooled for meta-analysis (16,804 patients), pooled sensitivity was 0.94 (95% CI 0.90–0.97); Specificity was 1.00 (95% CI 0.99–1.00) (Supplementary Fig. 1). The SROC Curve is shown in Supplementary Fig. 2. Diagnostic Odds Ratio (DOR) and positive likelihood ratio (LR+) were both high, and negative likelihood ratio (LR-) was less than one.

**Table 2:** Bivariate model summary statistics for negative CT head at any time point, and at <6 h of symptom onset, to exclude SAH.

Parameter	Estimate	95% LCI	95% UCI
Sensitivity (Overall)	0.942	0.901	0.967
Specificity (Overall)	1	0.999	1
DOR (Overall)	27833401.198	108702.542	7126771929.047
LR+ (Overall)	1617439.763	6747.473	387717200.015
LR- (Overall)	0.058	0.034	0.1
FPR (Overall)	0	0	0.001
Sensitivity (<6h)	0.995	0.941	1
Specificity (<6h)	1	0	1
DOR (<6h)	1.813	0	N/A
LR+ (<6h)	N/A	N/A	N/A
LR- (<6h)	0.005	0	0.061
FPR (<6h)	0	0	1
LP Sensitivity	0.997	0.712	1.00
LP Specificity	0.965	0.934	0.982
LP DOR	8471.15	64.05	1120386.70
LP LR+	28.80	14.93	55.36
LP LR-	0.003	0.00	0.412
LP FPR	0.035	0.018	0.066
I^2^ sensitivity (%)	0	I^2^ specificity (%)	0

DOR = Diagnostic Odds Ratio; LR+ = Likelihood Ratio for positive test results; LR- = Likelihood Ratio for negative test results; FPR = False positive rates.

**Fig. 3:**
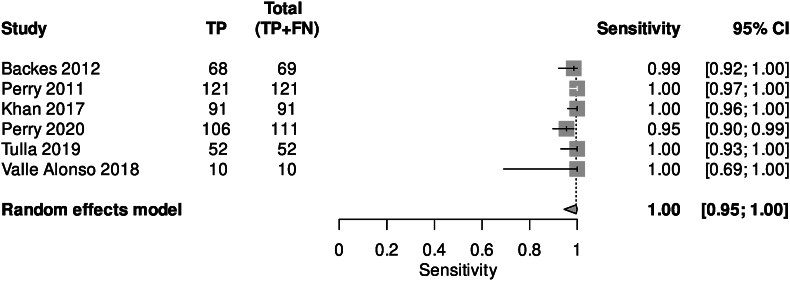
Pooled forest plot for CT head at <6 h of symptom onset.

The accuracy of CT at different time points is shown in Table 2. Six studies (9.5%) reported CT diagnostic accuracy within 6 h of symptom onset (3292 patients). Bivariable models were not converged so univariable results are presented. Among the 6 studies, pooled sensitivity was 0.995 (95% CI 0.941–1.000) (Fig. 3); Specificity was 1.00 (95% CI 0.00–1.00) (Supplementary Fig. 3). An SROC Curve could not be calculated due to lack of studies and model convergence (Supplementary Fig. 4). DOR was greater than one, LR + could not be calculated due to a lack of studies, and negative likelihood ratio (LR-) was less than one for the univariable model. Mean combined sensitivity was 0.988 (SD 0.018).

The accuracy of LP after negative intracranial CT is shown in Table 2. Thirty-four studies reported LP diagnostic accuracy. Among the thirty-four studies, pooled sensitivity was 0.99 (95% CI 0.71–1.00); Specificity was 0.97 (95% CI 0.93–0.98). The SROC Curve is shown in Supplementary Fig. 5.

Excluding selective populations (studies that only included CT negative patients who would be diagnosed by methods other than CT only), the pooled proportion of all SAH cases diagnosed by LP was 6.7% (95% CI 2.9–16.7). Using an estimated incidence of 4800 SAH diagnosed in the UK every year (incidence 8 per 100,000 per year- National Study of Subarachnoid haemorrhage) (RCS(Eng), 2006), we are able estimate the number of SAH that would be missed if LP was removed from the diagnostic pathway (as per recent NICE guidelines). Removing LP for any time pathway, 7% of SAH would not be identified, equating to a rate of 336 missed aneurysmal SAH every year. Removing LP if CT head is negative at 6 h, would lead to 0.5% of diagnosed aneurysmal SAH being missed, with 24 cases missed every year (95% CI 0–278 missed cases). If mean sensitivity was used, this would increase to 58 cases (0.12%, 95% CI 0–240 missed cases).

The results of the 3-step sensitivity analysis are shown in Supplementary Tables 6–14, and Supplementary Figs. 6–17. There was no difference in diagnostic accuracy of CT within 6 h when removing publications by the same author (N = 1), those with a high risk of bias (N = 1), or studies carried out in departments other than ED (N = 1). The diagnostic accuracy of CT for any time period increased when high risk of bias papers (N = 9) were removed, and decreased when non-ED studies (N = 8) were removed (Supplementary Tables 6–14). Diagnostic accuracy for LP improved when studies at high risk of bias were removed (N = 19), and when studies carried out in an ED department were removed (N = 15).

The assessment of bias for retrospective cohort studies, using the Newcastle-Ottawa Scale, is shown in Supplementary Fig. 18. The mean score (out of nine) for all studies was 5.7, and 36 (57.1%) studies were classified as high risk of bias. The differences in risk of bias scores, stratified by studies included in the NICE guidelines review, are shown in Supplementary Figs. 19–20. The studies included by NICE had a mean score of 6.7/9 (SD 2.30), and studies not included by NICE had a mean score of 5.36/9 (SD 2.39).

## 4 Discussion

This study presents a systematic review and meta-analysis of available literature to assess the diagnostic utility of CT head by timepoint in assessing SAH and the potential impact of omitting LP from the diagnostic pathway. Our data suggest a significant number of SAH would be missed if LP is omitted from the diagnostic pathway based on the current literature.

Crucially, our study demonstrates limitations in current evidence quality, which impacts the applicability of the recent NICE guidelines to clinical practice. Over half of included studies were at high risk of bias. The quality assessment highlights specific issues in the evidence base, such as a lack of clear inclusion criteria or reporting of absolute values to determine CT head sensitivity and specificity. Therefore, caution must be exercised before implementing such guidelines.

A missed SAH can be a catastrophic event with mortality reported as high as 10% (Takagi et al., 2018a) and associates with an increased risk of death and disability (Kowalski et al., 2004). Re-bleeding also occurs in approximately 8–23% patients within the first 72 h after ictus (Calviere et al., 2022a; Stienen et al., 2018). Our study showcases that, overall, very few cases of SAH would be missed if a ‘6-h rule’ was implemented, however, the personal, healthcare and economic ramifications for the number missed would likely be severe. This is also likely an underestimate of the potential missed cases when considering studies that have reported a higher SAH incidence than used in our calculations (Pobereskin, 2001). Furthermore, the UK population at the time of study publication was significantly less, which could raise the incidence to 5400, and missed cases to 30 per year. The risks of re-bleeding are also likely higher in patients discharged with a false negative CT head (Calviere et al., 2022b; Starke et al., 2011; Oh et al., 2018), owing to likely delays in a second presentation to hospital. Whilst there are compelling arguments to support this guideline from an efficiency and economic, these arguments must be balanced with the real implications for patients with 10.13039/100005812SAH but a negative CT head. The use of LP has been indicated in this review for this patient group, and thus a value judgement determining the acceptable level of risk must be defined by policymakers.

Whilst LP can help identify SAH, it is not without risks, and the risk of traumatic tap, failed procedure, and post-procedure headache requiring re-admission, remains significant (Ahmed et al., 2006). Further, LP is time consuming, and resource intensive to health systems, with many patients requiring admission to hospital as an inpatient for an LP, producing additional costs (Wu et al., 2016).

The NICE guidelines advocate that a CT head can exclude a SAH if performed within 6 h of symptoms when reported by a radiologist (NICE, 2022). Yet, there is evidence demonstrating that either neuroradiologists or radiologists who frequently interpret brain images are necessary to achieve this (Walton et al., 2022). Out of six studies that reported diagnostic accuracy <6 h after symptom onset only one study including CT scans exclusively reported by neuroradiologists. Many studies showcase real-life practice, where many centres do not have access to on-site reporting neuroradiologists, or board certified radiologists, in contrast to research studies (Schofield et al., 2004). This distinction must also be made and highlighted as it impacts the applicability of the NICE guidelines (NICE, 2022), as this service is not available in all centres.

Furthermore, five out of six studies in this group were produced solely in emergency departments. The lack of inclusion of neurosurgical teams, who are a key stakeholder involved in tertiary treatment of patients with SAH, may also be significant. Many neurosurgeons advocate for LP to remain in the diagnostic pathway based on institutional experience, which in contrast to ED studies, comes at the end of the diagnostic pathway (Martin et al., 2015). ED physicians review the undifferentiated acute headache, whilst neurosurgeons are exposed to a focused patient population with a higher propensity towards a positive CT head or positive lumbar puncture. Both of these key stakeholders hold important perspectives which should inform policymaking in the absence of high quality data.

When released in 2022, the guidelines made the recommendation that, if a CT head was negative within 6 h of symptom onset, and had been reported by a radiologist, an LP should not be offered routinely (NICE, 2022). We endeavoured to include all previously published literature in our evidence synthesis. Nonetheless, this small number of studies (N = 6) could be considered low to inform policy, with few included being prospective, multi-centre studies with low risk of bias. When considering the potential consequences of removing a diagnostic test from a pathway of critical illness, policymakers must be confident that the evidence-base and subsequent decision-making holds patient safety central. Considering the evidence of CT negative SAH identified on LP, presently CT head is not able to diagnose all patients presenting with SAH.

Lumbar puncture adds additional cost to the work-up of a patient presenting to A&E with acute headache (Giamberardino et al., 2020), and as such received dedicated economic analysis in the 2022 NICE Guidelines. A lumbar puncture was estimated to cost approximately £610 due to non-elective short stay admission costs. The challenge is balancing the estimation of cost of a lumbar puncture with the cost of a missed diagnosis of SAH, of which there are no formal economic evaluations. Cost-of-illness studies indicate the high economic burden of aneurysmal SAH (Rivero-Arias et al., 2010; Modi et al., 2019), and poorer functional outcomes and SAH complications increase costs, resulting from diagnostic delay or treatment delay (Takagi et al., 2018b).

In the UK, the cost of performing lumbar punctures on all suspected SAH with a negative CT head would be £14,640. Pooled sensitivity for CT head under 6 h in our study was 0.995, meaning 24 cases of CT head negative SAH would be missed. This differed to the NICE estimation. Using £23,974 as a cost-per-patient (Rivero-Arias et al., 2010), and acknowledging this is an average estimation for the typical SAH patient, the cost to UK health systems in economic terms per year would be £575,376. However, one would expect a higher cost per-annum in patients in those missed with the CT head strategy alone, due to the diagnostic delay (if they successfully represent to hospital), and increasing incidence of SAH compared to the latest incidence estimation.

The results of our analysis are similar to a large, prospective cohort study, that demonstrated high sensitivity for CT head at <6 h from symptom onset (Perry et al., 2011; Dubosh et al., 2016). The findings, while similar to a published review include a robust quality assessment, in addition to the sensitivity and specificity values of LP (Carpenter et al., 2016). This study adds that a significant number of SAH are still diagnosed by LP (6.7%), constituting a significant contribution to SAH diagnosis.

This study has several limitations. Only a small number of included studies were prospective and multi-centre. The high risk of bias rating for most studies reflects critical flaws in methodology, and reporting. Of 63 studies, only half had data that could be pooled for meta-analysis, and less than 10% included data on timing <6 h. Many studies included time ranges, and largely defined timing as starting from symptom onset, but did not provide sufficient data to calculate sensitivity and specificity, precluding them for inclusion in pooled analysis.

Our review process also carries several limitations. Firstly, we excluded studies published before 2000, excluding at least one significant paper (van der Wee et al., 1995), although all studies identified by screening abstracts published between 1980 and 2000 reported sensitivity at <12 h or <24 h (Sames et al., 1996; Sidman et al., 1996), and not the primary outcome of <6 h. In addition, we only included studies published in English or with a clear translation available (Valle Alonso et al., 2018). In addition, our estimate of missed SAH cases relies on the assumption that all 4800 SAH present and have a CT head within 6h of ictus, which does not reflect real practice, with increasing clinical suspicion of SAH usually meriting an earlier scan. In our study, these populations accounted for a quarter (26.7%) of the total possible SAH population. In addition, this figure assumes this would be implemented as a stand-alone policy without specialist advice that LP was contraindicated or not required.

Due to lack of equipoise precluding an RCT, Instead, we advocate the need for a multi-centre, prospective study and evidence review including multi-disciplinary stakeholders, to best advocate for and solve this important unsolved question. A final limitation is that ultimately, this study confirms results of what is already generally known from previously published studies (Dubosh et al., 2016; Claassen et al., 2022).

This study has implications for clinical practice. It adds a critical appraisal of the current NICE guidelines and its informant literature. The studies that compose and influence the latest guideline have all been synthesised, with a lack of studies confirming the absolute efficacy of CT head at <6 h after symptom onset. Whether the existing evidence is sufficient to justify a change in practice, remains the decision of treating clinicians.

## 5 Conclusion

CT head appears to be highly sensitive at excluding SAH <6 h from symptom onset. High quality, prospective data is required to further established the utility of early (<6 h) negative CT head. We recommend that if there is strong clinical suspicion of SAH (from primary or secondary teams), yet CT head is negative within 6 h of symptom onset, that a LP be performed.

## Patient consent for publication

Not required.

## Data sharing statement

All study data is available by contacting the corresponding authors.

## Author contributions

PJH, AH, RAT, PCW, RV, and NG conceived the study. CSG and JGH drafted the initial study protocol. CSG, RM, KSL, and MA collected data for the study. CSG and KSL conducted statistical analysis. CSG, JGH, AMA, JOE, and OM drafted the first manuscript draft. JGH, SCW, and JPF reviewed the first manuscript draft, and provided input and suggestions. NG, RV, PCW, RAT, AH, and PJH reviewed the second iteration of the manuscript, and provided senior guidance and support throughout. PJH is the senior author. All authors proofread and approved the final manuscript.

## Funding

This research received no specific grant from any funding agency in the public, commercial or not-for-profit sectors.

## Declaration of competing interest

PW is the current president of the Society of British Neurological Surgeons (SBNS). NG is the current NICE coordinator for the SBNS. RV is the current Chair of the British Neurovascular Group (BNVG). PH is Meetings and Communications Secretary of the SBNS. PJH, AEH, RATR are council members of the SBNS.
