# Supplementary material for: Diagnosis of subarachnoid haemorrhage: Systematic evaluation of CT head diagnostic accuracy and comparison with the 2022 NICE guidelines
Source: Brain Spine. 2025 Feb 4;5:104200. doi: 10.1016/j.bas.2025.104200 (PMC11872663; doi:10.1016/j.bas.2025.104200)
Supplement: Multimedia component 1 [file mmc1.docx]

**Supplementary Tables**

Supplementary Table 1. Medline search strategy (Search performed 27^th^ November 2022).

| Search | Query | Results (27/11) |
| --- | --- | --- |
| 1 | exp subarachnoid hemorrhage/ | 23540 |
| 2 | (subarachnoid hemorrhag* or subarachnoid haemorrhag*).mp | 33098 |
| 3 | 1 or 2 | 33098 |
| 4 | (Lumbar puncture).mp | 9030 |
| 5 | exp Spinal Puncture/ | 6688 |
| 6 | 4 or 5 | 12775 |
| 7 | 3 and 6 | 3301 |
| 8 | Limit 7 to humans | 2692 |
| 9 | Limit 8 to English language | 2242 |

Supplementary Table 2. CINAHL Plus search strategy (Search performed 27^th^ November 2022).

| Search | Query | Results (27/11) |
| --- | --- | --- |
| 1 | (MH "Subarachnoid Hemorrhage") | 5278 |
| 2 | (subarachnoid hemorrhag* or subarachnoid haemorrhag*) | 7,546 |
| 3 | S1 or S2 | 7546 |
| 4 | (Lumbar puncture) | 3148 |
| 5 | (MH "Spinal Puncture") | 2325 |
| 6 | S4 or S5 | 3,973 |
| 7 | S3 and S6 | 257 |

Supplementary Table 3. Cochrane library search strategy (Search performed 27^th^ November 2022).

| Search | Query | Results (27/11) |
| --- | --- | --- |
| 1 | “subarachnoid hemorrhage” or “subarachnoid haemorrhage” | 2328 |
| 2 | Lumbar Puncture | 1519 |
| 3 | #1 AND #2 | 42 |

Supplementary Table 4. Embase search strategy (Search performed 27^th^ November 2022).

| Search | Query | Results (27/11) |
| --- | --- | --- |
| 1 | exp subarachnoid hemorrhage/ | N/A |
| 2 | (subarachnoid hemorrhag* or subarachnoid haemorrhag*).mp | N/A |
| 3 | 1 or 2 | N/A |
| 4 | (Lumbar puncture).mp | N/A |
| 5 | exp Lumbar Puncture/ | N/A |
| 6 | 4 or 5 | N/A |
| 7 | 3 and 6 | N/A |
| 8 | Limit 7 to humans | N/A |
| 9 | Limit 8 to English language | 1436 |

Supplementary Table 5. PICOS inclusion criteria

| **Review Question** | In patients with suspected SAH with negative intracranial imaging, how many true cases are identified on a subsequent Lumbar Puncture (LP)? | |
| --- | --- | --- |
| **Population** | Adults ≥18 years with clinically suspected SAH, with negative CT imaging performed within any time frame (e.g 6 hours, 12 hours, 24 hours). | |
| **Intervention** | Lumbar Puncture (CSF analysis) | |
| **Comparator** | No Lumbar puncture or CT positive scan | |
| **Outcomes** | **Primary** | **Secondary** |
|  | Sensitivity & Specificity of 6 hour CT | Identify how many Lumbar punctures conducted as second line investigation result in a SAH diagnosis overall, as a percentage of all LPs conducted |
|  | Sensitivity of CT performed at any time period | Within these studies, how many were diagnosed by CT imaging, and how many by Lumbar Puncture. |
|  | Identify number (%) of LP that when performed after negative CT head, identify a SAH successfully | Quality of all included studies |
| **Setting** | Studies taking place in any emergency, neurology, neurosurgery, biochemistry or pathology department | |
| **Study design** | Phase 3 trials, prospective case series and cohort studies with >1 adult patient | |
| **Follow-up** | Report end diagnostic result of admission | |

Supplementary Table 6. Sensitivity Analysis parameters for CT head at any time point, for studies at high risk of bias (n=9).

| **Parameter** | **Estimate** | **95% LCI** | **95% UCI** |
| --- | --- | --- | --- |
| Sensitivity | 0.875 | 0.781 | 0.932 |
| Specificity | 1 | 0.985 | 1 |
| DOR | 1189246 | 445.575 | 3.17E+09 |
| LR+ | 148259 | 56.169 | 3.91E+08 |
| LR- | 0.125 | 0.069 | 0.225 |
| FPR | 0 | 0 | 0.015 |

Supplementary Table 7. Sensitivity Analysis parameters for CT head at any time point, for studies at low of bias (n=11).

| **Parameter** | **Estimate** | **95% LCI** | **95% UCI** |
| --- | --- | --- | --- |
| Sensitivity | 0.961 | 0.91 | 0.984 |
| Specificity | 1 | 0.945 | 1 |
| DOR | 1872337 | 311.904 | 1.12E+10 |
| LR+ | 72136.07 | 16.466 | 3.16E+08 |
| LR- | 0.039 | 0.016 | 0.092 |
| FPR | 0 | 0 | 0.055 |

Supplementary Table 8. Sensitivity Analysis parameters for CT head at any time point, for studies performed in ED departments (n=12).

| **Parameter** | **Estimate** | **95% LCI** | **95% UCI** |
| --- | --- | --- | --- |
| Sensitivity | 0.926 | 0.843 | 0.967 |
| Specificity | 1 | 0.994 | 1 |
| DOR | 2902280 | 1806.51 | 4.66E+09 |
| LR+ | 215874.8 | 151.895 | 3.07E+08 |
| LR- | 0.074 | 0.034 | 0.162 |
| FPR | 0 | 0 | 0.006 |

Supplementary Table 9. Sensitivity Analysis parameters for CT head at any time point, for studies performed in non-ED departments (n=8).

| **Parameter** | **Estimate** | **95% LCI** | **95% UCI** |
| --- | --- | --- | --- |
| Sensitivity | 0.949 | 0.874 | 0.98 |
| Specificity | 1 | 0.385 | 1 |
| DOR | 547399.4 | 10.186 | 2.94E+10 |
| LR+ | 27993.5 | 0.592 | 1.32E+09 |
| LR- | 0.051 | 0.02 | 0.13 |
| FPR | 0 | 0 | 0.615 |

Supplementary Table 10. Sensitivity Analysis parameters for CT head at <6hrs, with studies at high risk of bias (n=1) removed.

| **Parameter** | **Estimate** | **95% LCI** | **95% UCI** |
| --- | --- | --- | --- |
| Sensitivity | 0.99 | 0.942 | 0.998 |
| Specificity | 1 | 0 | 1 |
| DOR | 6.95E+20 | 0 | Inf |
| LR+ | Inf | NA | NA |
| LR- | 0.01 | 0.002 | 0.06 |
| FPR | 0 | 0 | 1 |

Supplementary Table 11. Sensitivity Analysis parameters for CT head at <6hrs, with studies not from an ED department (n=1) removed.

| **Parameter** | **Estimate** | **95% LCI** | **95% UCI** |
| --- | --- | --- | --- |
| Sensitivity | 1 | 0.138 | 1 |
| Specificity | 1 | 0 | 1 |
| DOR | 2.74E+19 | 0 | Inf |
| LR+ | 4.5E+15 | 0 | Inf |
| LR- | 0 | 0 | 6.244 |
| FPR | 0 | 0 | 1 |

Supplementary Table 12. Sensitivity Analysis parameters for CT head at <6hrs, with studies from the same authorship group (n=1) removed.

| **Parameter** | **Estimate** | **95% LCI** | **95% UCI** |
| --- | --- | --- | --- |
| Sensitivity | 0.992 | 0.936 | 0.999 |
| Specificity | 1 | 0 | 1 |
| DOR | 9.03E+20 | 0 | Inf |
| LR+ | Inf | NA | NA |
| LR- | 0.008 | 0.001 | 0.067 |
| FPR | 0 | 0 | 1 |

Supplementary Table 13. Sensitivity Analysis parameters for LP, with studies with studies at high risk of bias (N=19) removed.

| **Low risk of bias (N=15)** | | | | **High risk of bias (N=19)** | | | |
| --- | --- | --- | --- | --- | --- | --- | --- |
| **Parameter** | **Estimate** | **95% LCI** | **95% UCI** | **Parameter** | **Estimate** | **95% LCI** | **95% UCI** |
| Sensitivity | 0.992 | 0.155 | 1 | Sensitivity | 0.983 | 0.586 | 1 |
| Specificity | 0.981 | 0.958 | 0.992 | Specificity | 0.931 | 0.846 | 0.971 |
| DOR | 6286.893 | 8.863 | 4459487 | DOR | 761.961 | 13.864 | 41875.91 |
| LR+ | 52.18 | 23.304 | 116.836 | LR+ | 14.189 | 6.04 | 33.333 |
| LR- | 0.008 | 0 | 5.215 | LR- | 0.019 | 0 | 0.711 |
| FPR | 0.019 | 0.008 | 0.042 | FPR | 0.069 | 0.029 | 0.154 |

Supplementary Table 14. Sensitivity Analysis parameters for LP, with studies in an ED department (N=15) removed.

| **Non-ED studies (N=18)** | | | | **ED Department studies (N=16)** | | | |
| --- | --- | --- | --- | --- | --- | --- | --- |
| **Parameter** | **Estimate** | **95% LCI** | **95% UCI** | **Parameter** | **Estimate** | **95% LCI** | **95% UCI** |
| Sensitivity | 0.998 | 0.101 | 1 | Sensitivity | 0.965 | 0.461 | 0.999 |
| Specificity | 0.969 | 0.917 | 0.989 | Specificity | 0.963 | 0.912 | 0.985 |
| DOR | 16396.98 | 3.362 | 79964393 | DOR | 708.625 | 19.227 | 26116.64 |
| LR+ | 32.578 | 11.789 | 90.025 | LR+ | 25.788 | 10.626 | 62.583 |
| LR- | 0.002 | 0 | 8.993 | LR- | 0.036 | 0.001 | 1.039 |
| FPR | 0.031 | 0.011 | 0.083 | FPR | 0.037 | 0.015 | 0.088 |
