# Supplementary material for: Diagnosis of subarachnoid haemorrhage: Systematic evaluation of CT head diagnostic accuracy and comparison with the 2022 NICE guidelines
Source: Brain Spine. 2025 Feb 4;5:104200. doi: 10.1016/j.bas.2025.104200 (PMC11872663; doi:10.1016/j.bas.2025.104200)

**Supplementary Figures**

Supplementary Figure 1. Specificity forest plot for CT head at any time point.


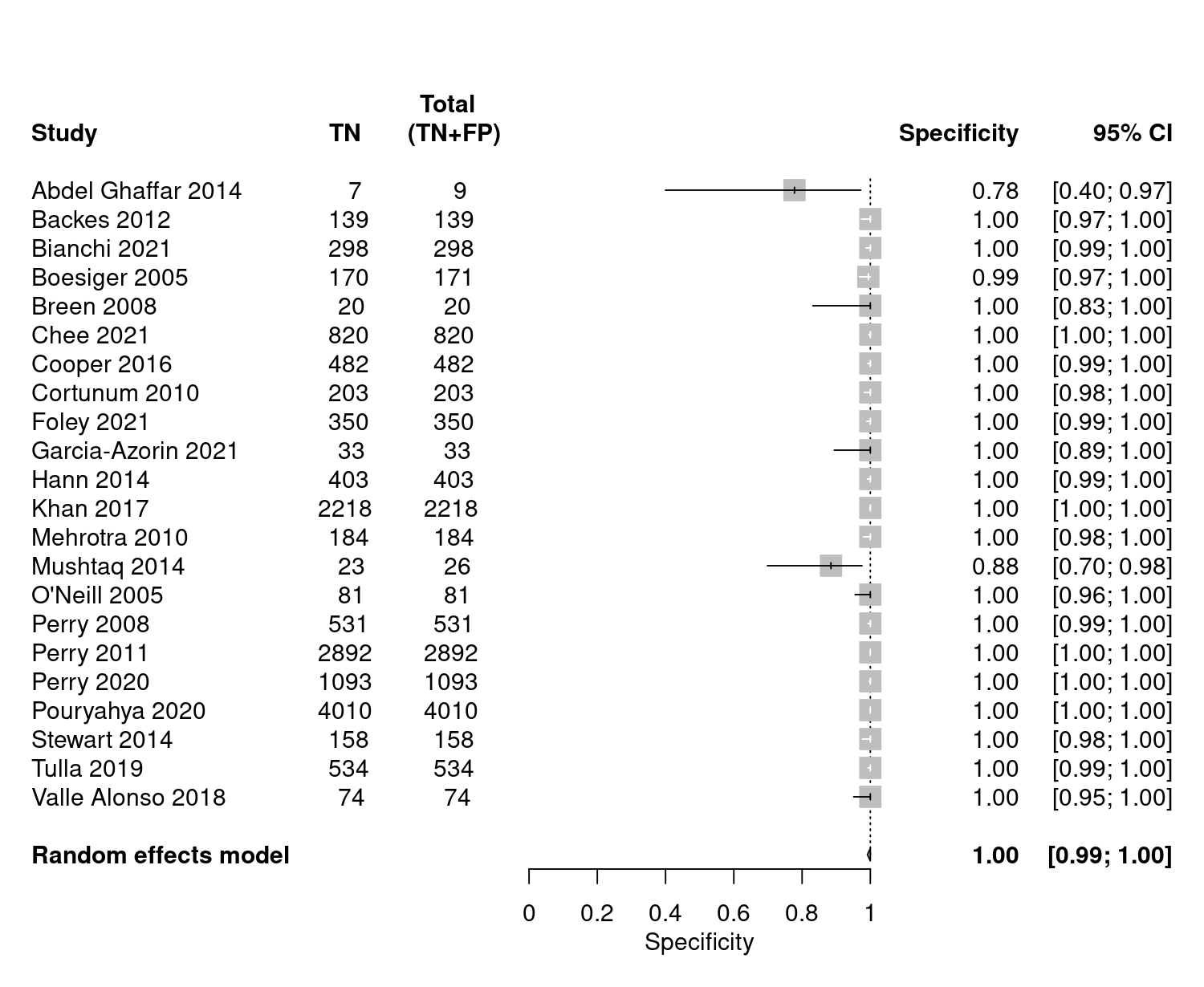


Supplementary Figure 2. Summary of receiver operating characteristics (SROC) curve of CT head diagnostic accuracy at any time point.


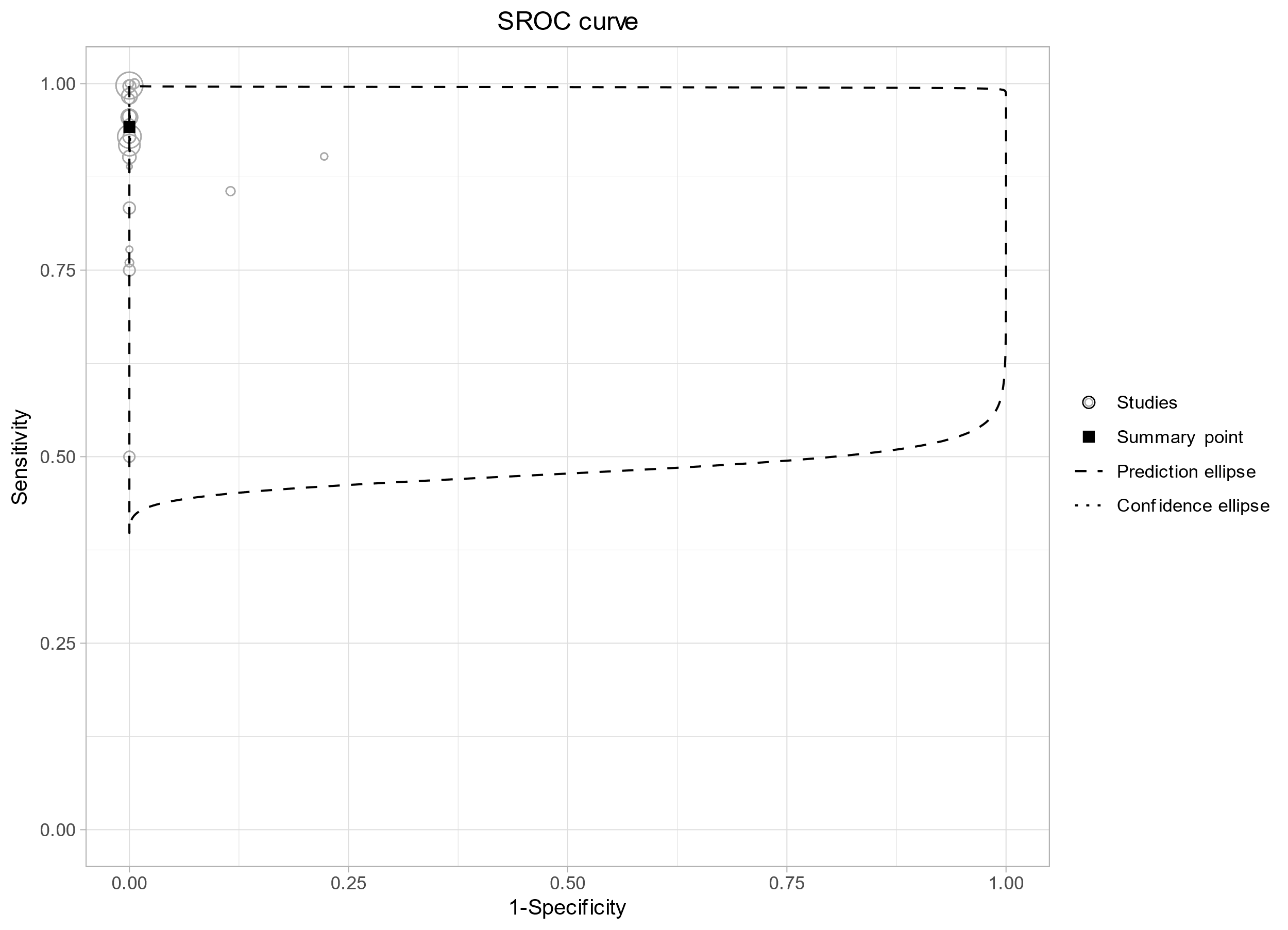


Supplementary Figure 3. Specificity forest plot for CT head at < 6 hours after symptom onset.

**
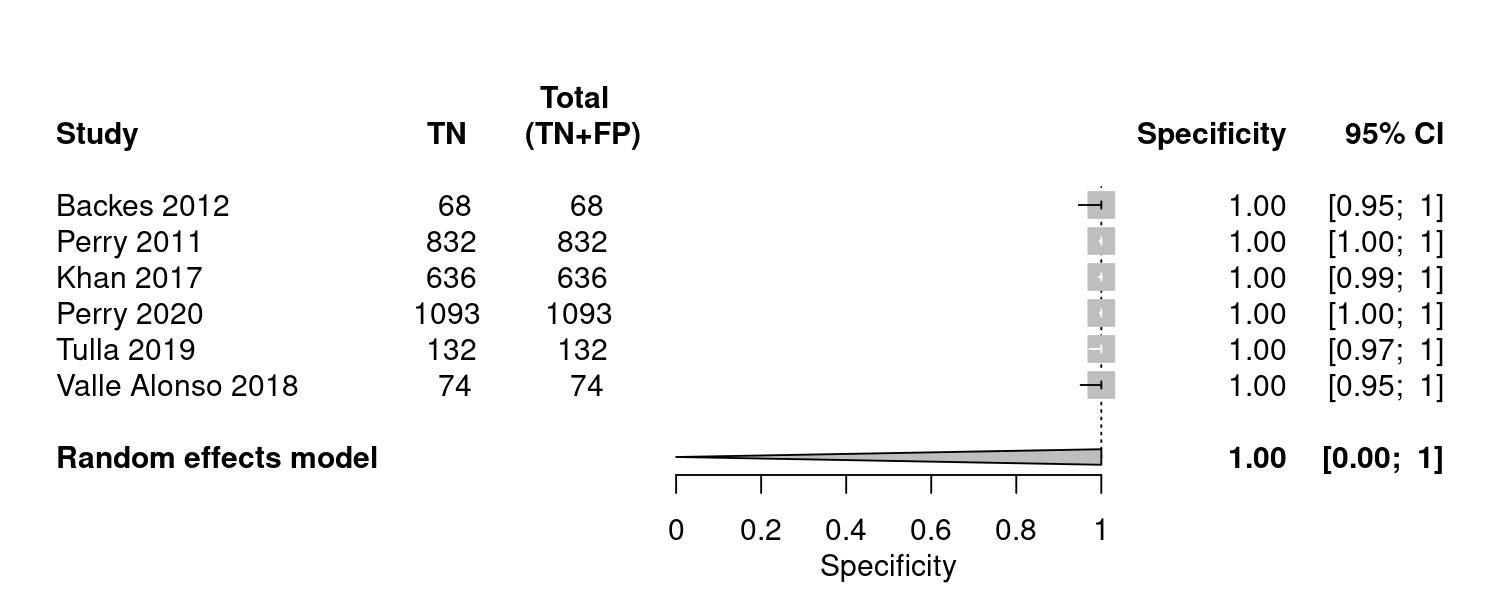
**

Supplementary Figure 4. ROC plane diagram of pooled studies for CT head within 6 hours of symptom onset, with 95% Confidence Interval bars.


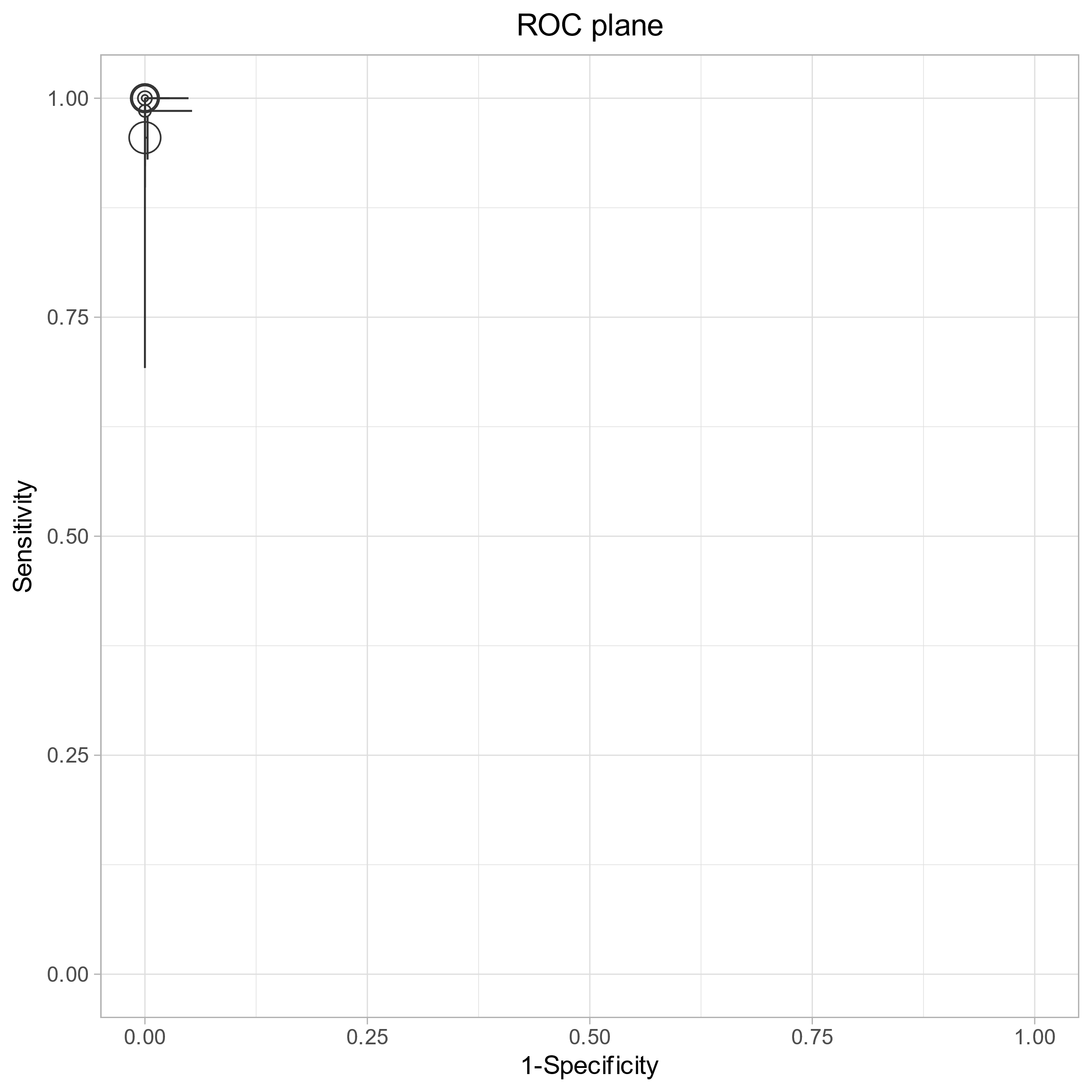


Supplementary Figure 3. SROC curve of LP diagnostic accuracy (34 studies).


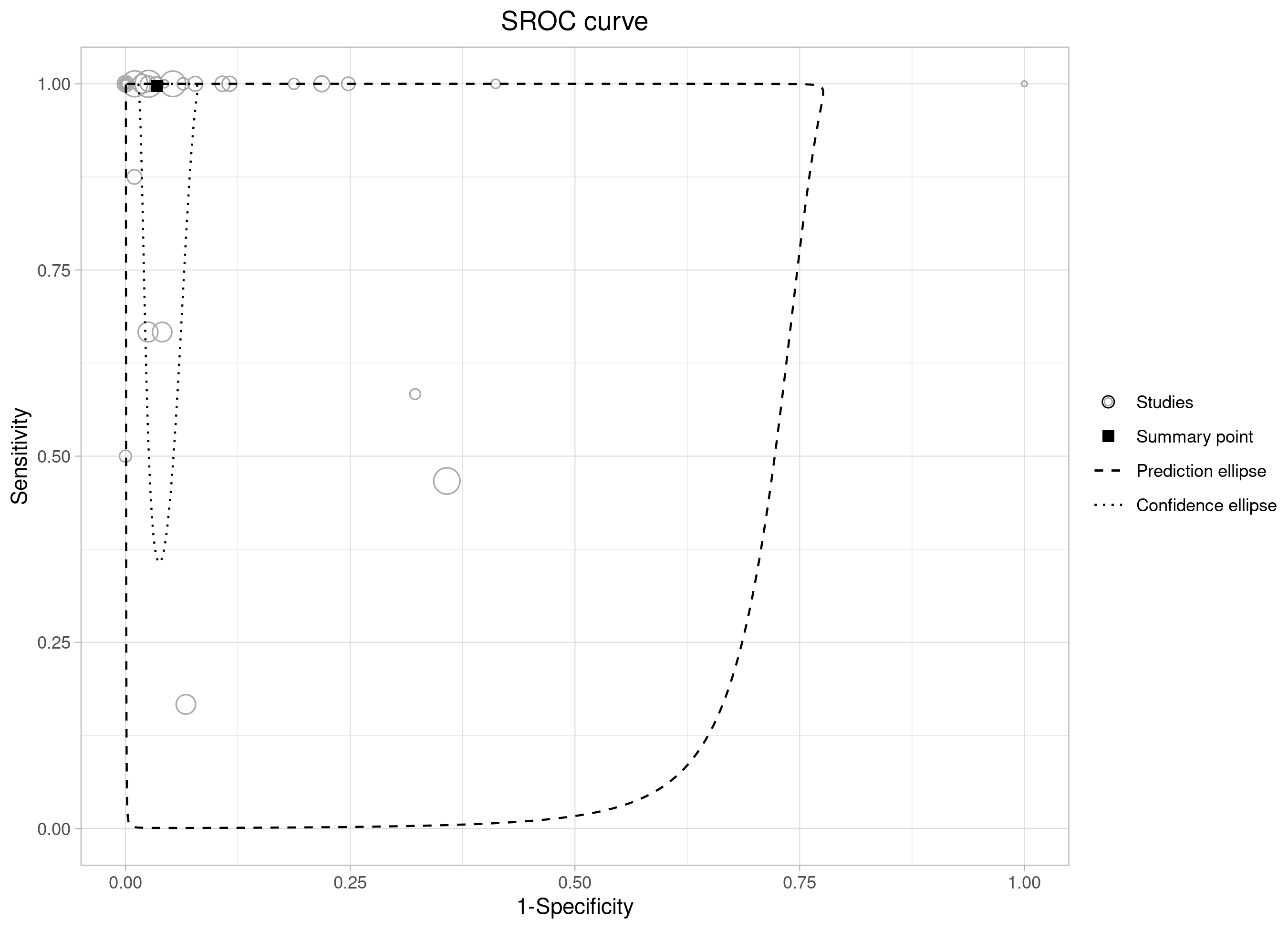


Supplementary Figure 4. Sub group analysis Sensitivity forest plot of studies for CT head at any time point, stratified by risk of bias.

**
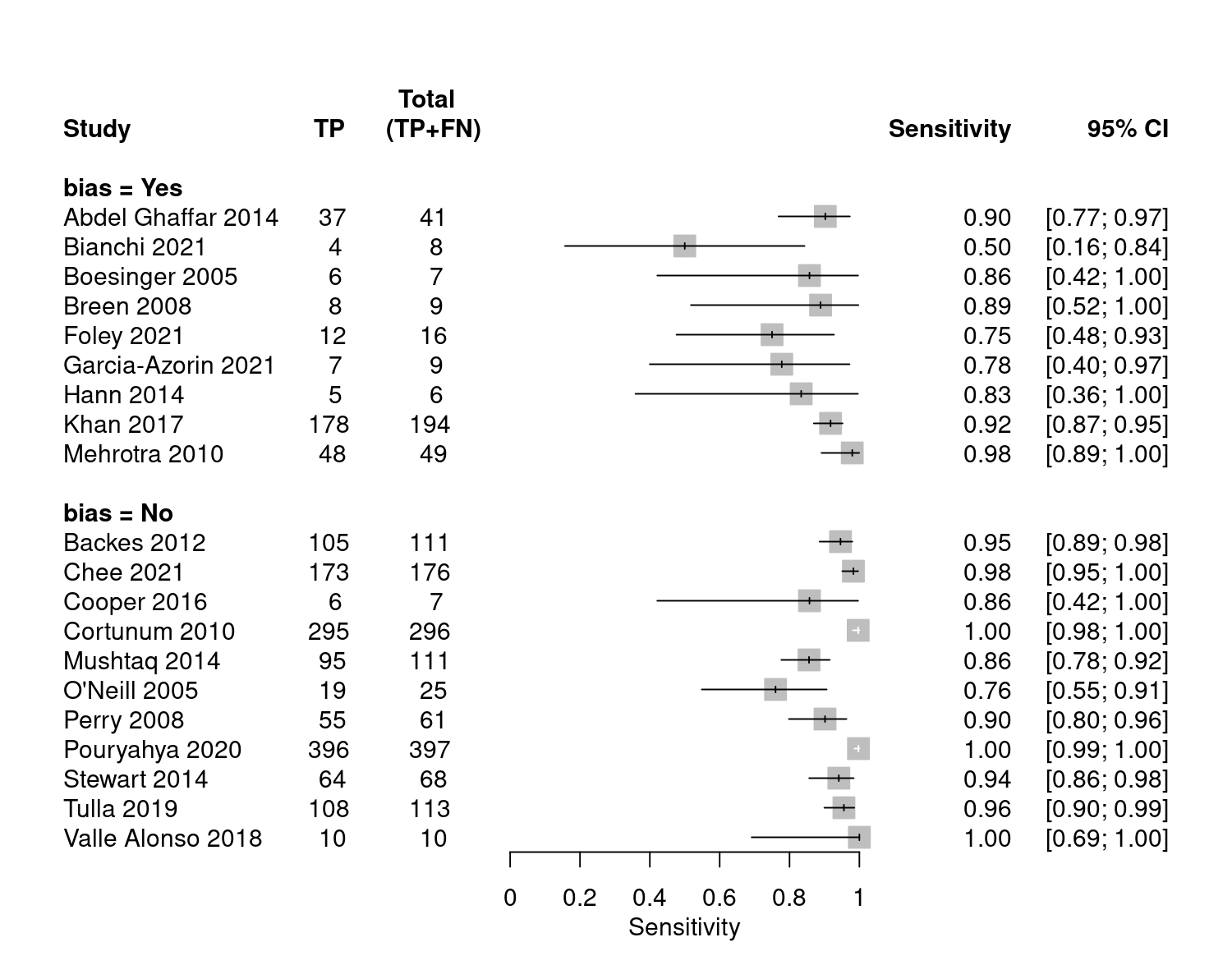
**

Supplementary Figure 5. Sub group analysis Specificity forest plot of studies for CT head at any time point, stratified by risk of bias.

**
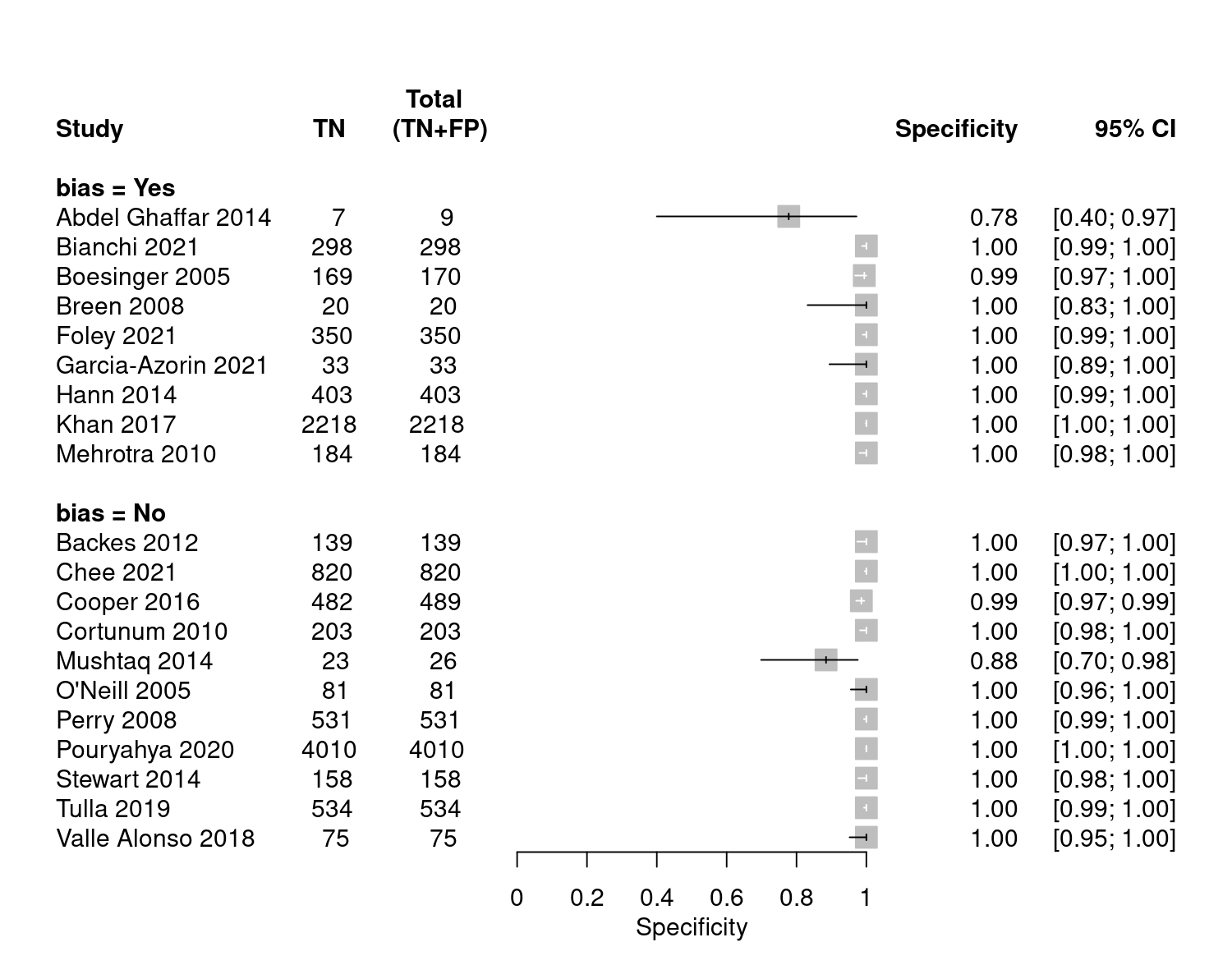
**

Supplementary Figure 6. SROC curve of subgroup analysis for CT head at any time point, comparing diagnostic accuracy of High (Yes) and Low (No) risk of bias.

**
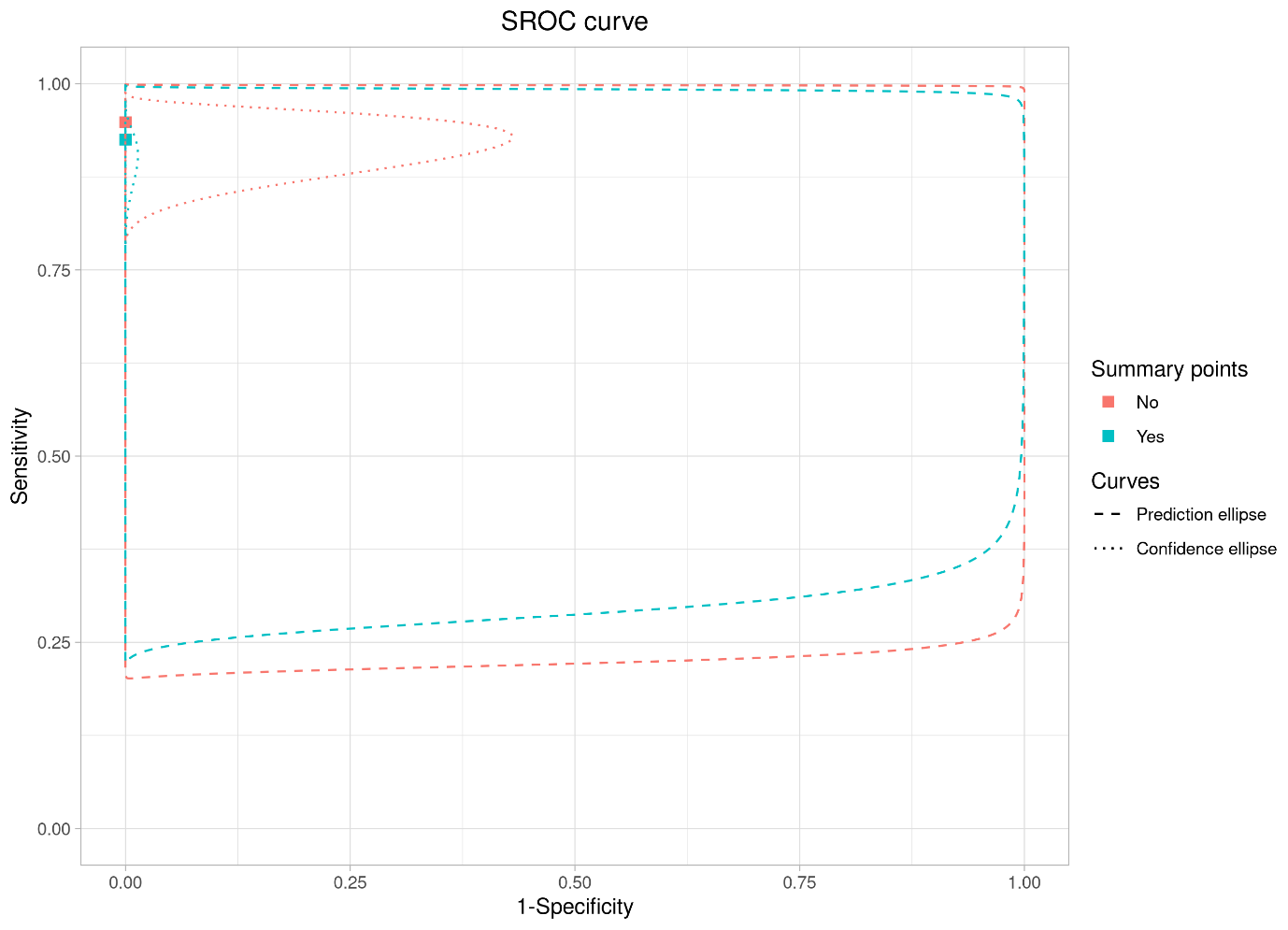
**

Supplementary Figure 7. SROC curve of subgroup analysis for CT head at any time point, comparing diagnostic accuracy of ED (Yes) and Non-ED (No) manuscripts.


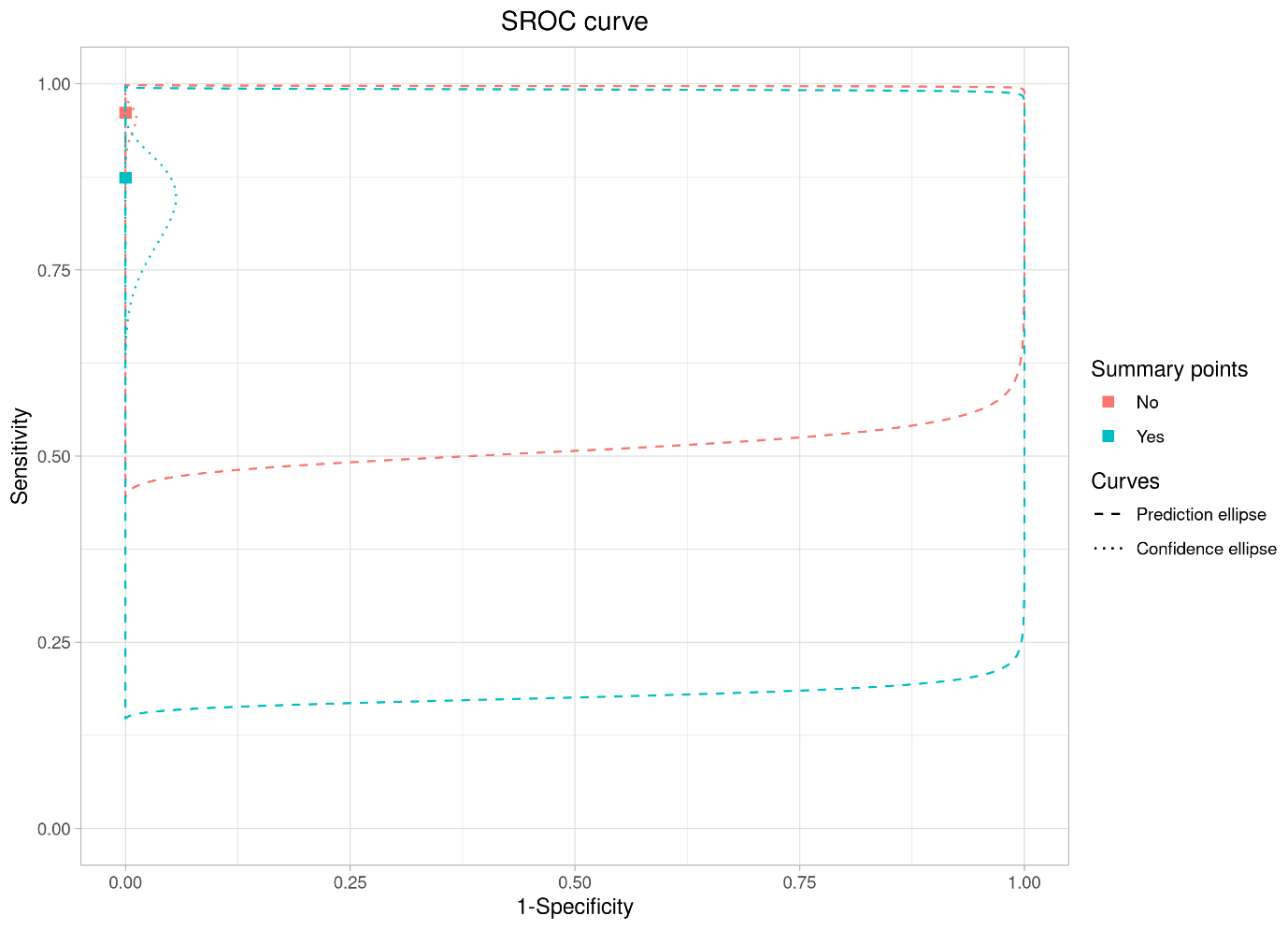


Supplementary Figure 8. Sub group analysis Sensitivity forest plot of studies for CT head at <6hrs, stratified by risk of bias.


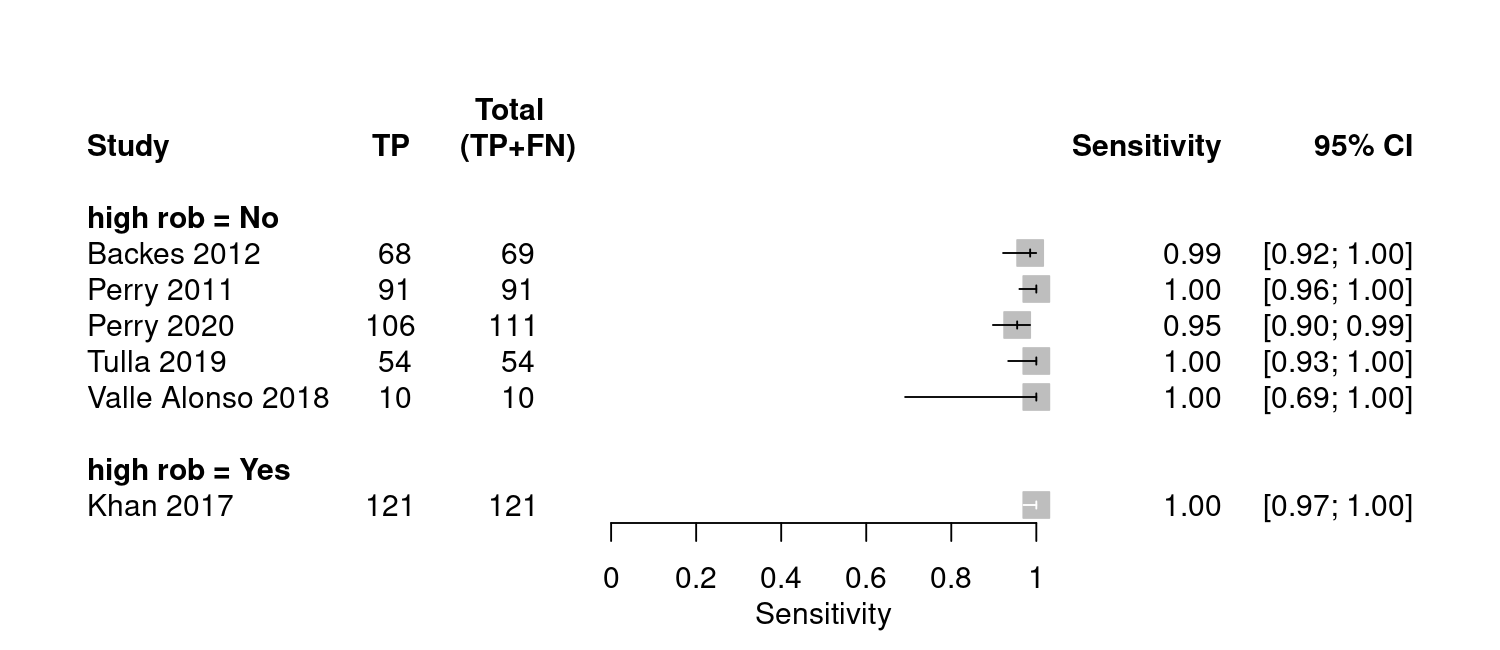


Supplementary Figure 9. Sub group analysis Sensitivity forest plot of studies for CT head at <6hrs, stratified by ED study authorship.


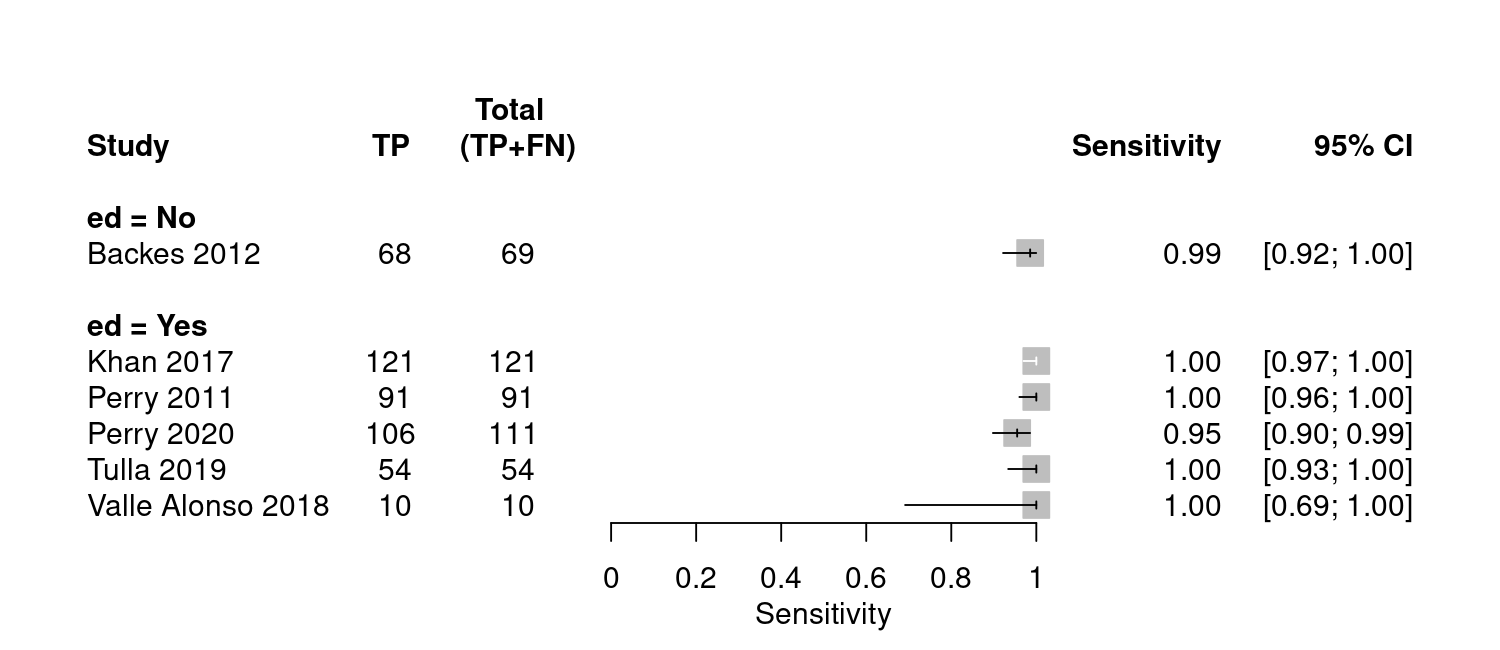


Supplementary Figure 9. Sensitivity analysis Sensitivity forest plot of studies for CT head at <6hrs, with high risk of bias studies (n=1) removed.


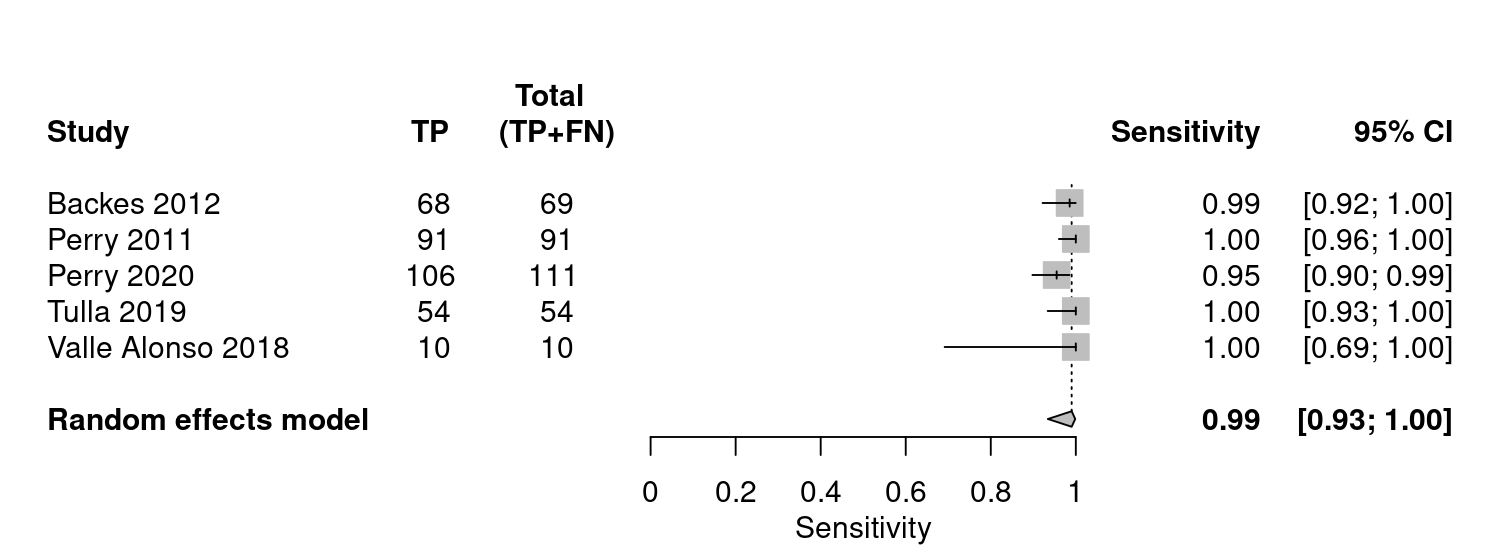


Supplementary Figure 10. Sensitivity analysis Specificity forest plot of studies for CT head at <6hrs, with high risk of bias studies (n=1) removed.


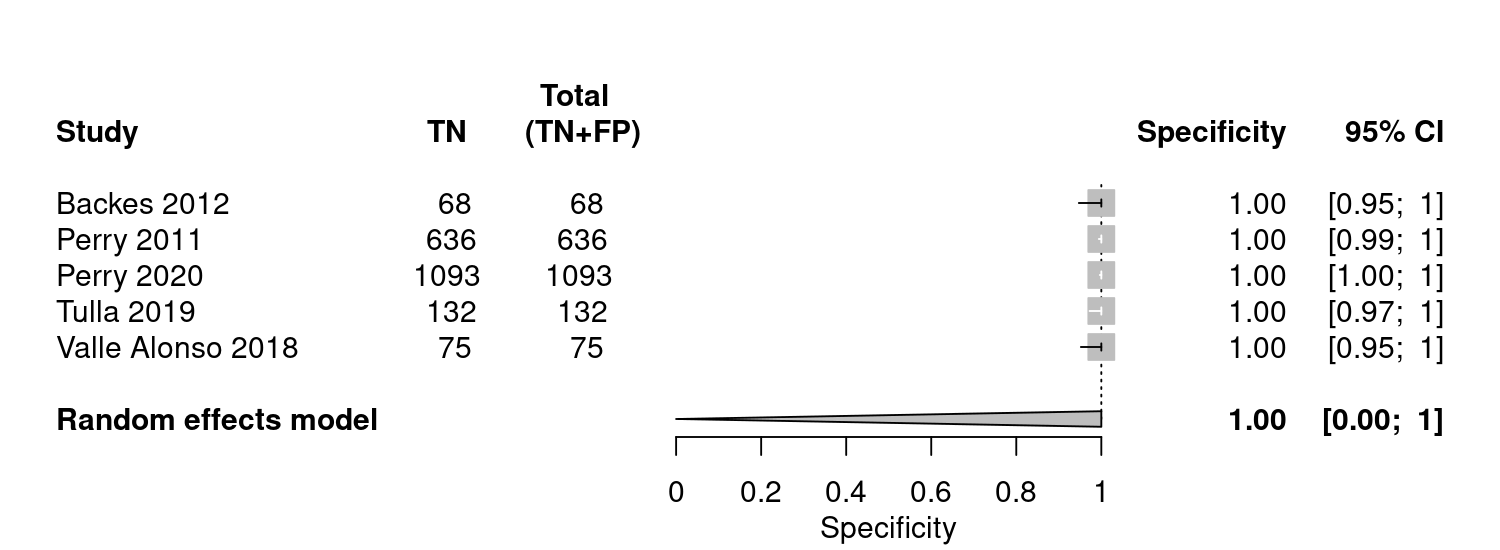


Supplementary Figure 11. Sensitivity analysis Specificity forest plot of studies for CT head at <6hrs, with Non-ED studies (n=1) removed.


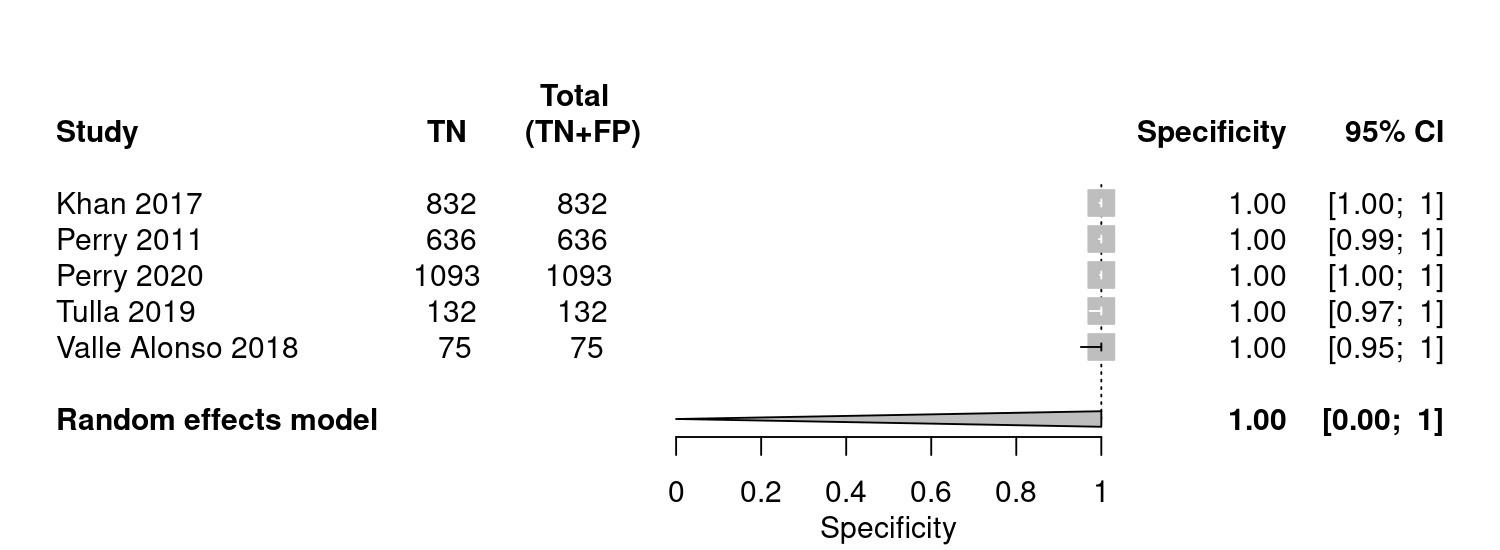


Supplementary Figure 12. Sensitivity analysis Sensitivity forest plot of studies for CT head at <6hrs, with publications from the same authorship group (n=1) removed.


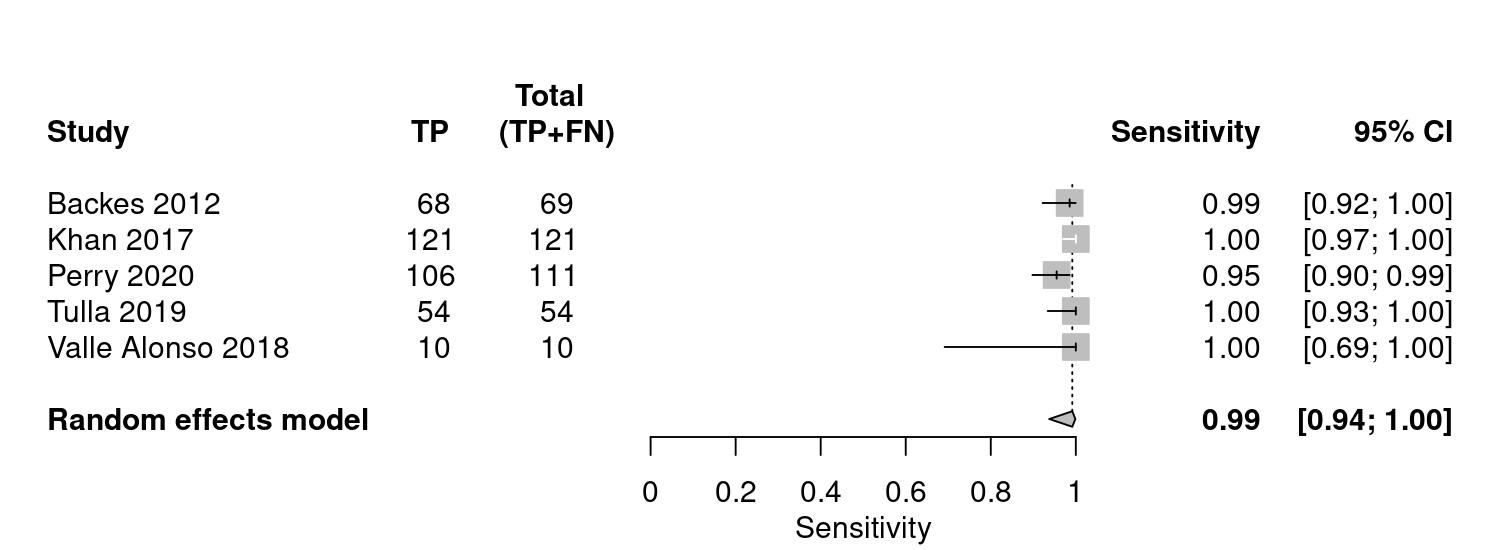


Supplementary Figure 13. Sensitivity analysis Specificity forest plot of studies for CT head at <6hrs, with publications from the same authorship group (n=1) removed.


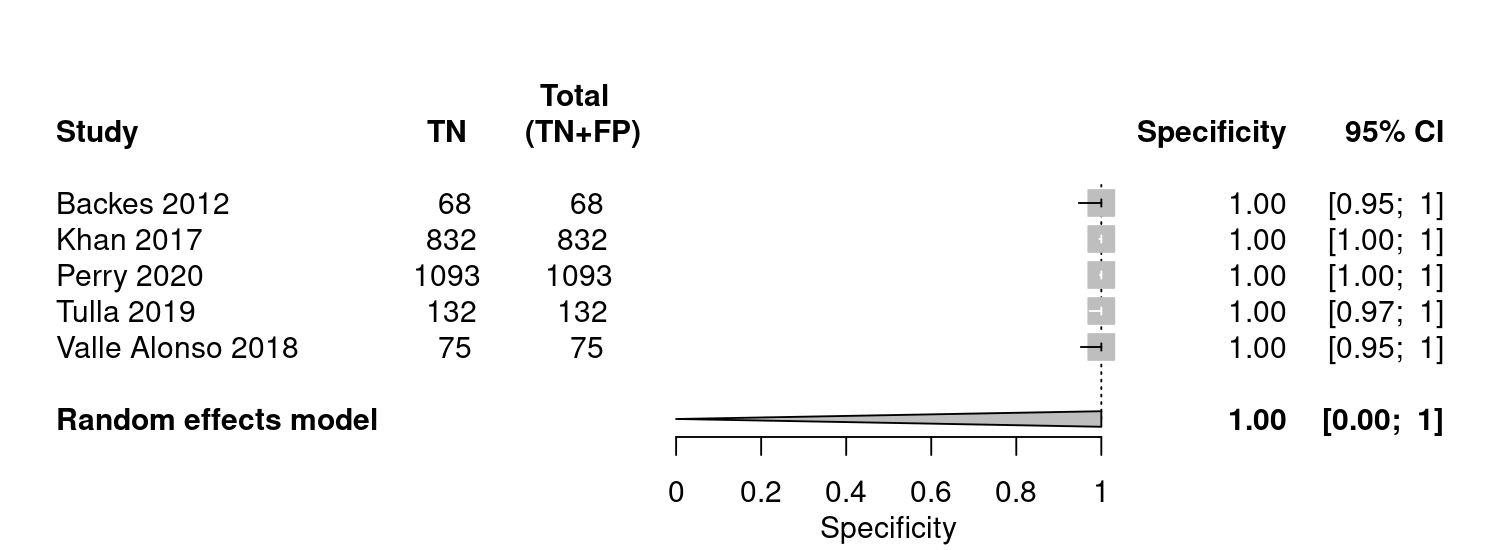


Supplementary Figure 14. SROC curve of subgroup analysis for LP accuracy, comparing diagnostic accuracy of High risk of bias (Yes) and Low risk of bias (No) manuscripts.


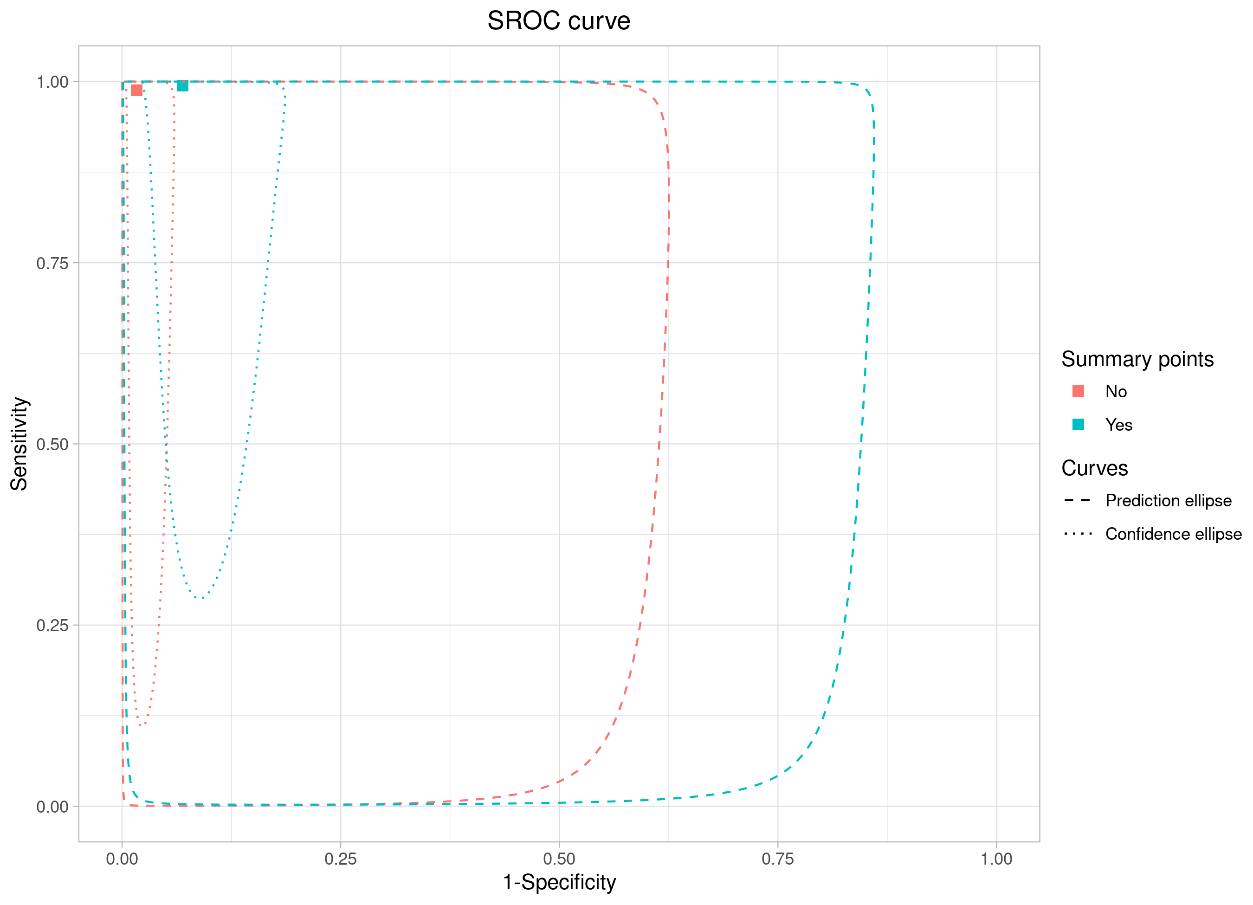


Supplementary Figure 15. SROC curve of subgroup analysis for LP at any time point, comparing diagnostic accuracy of ED (Yes) and Non-ED (No) manuscripts.


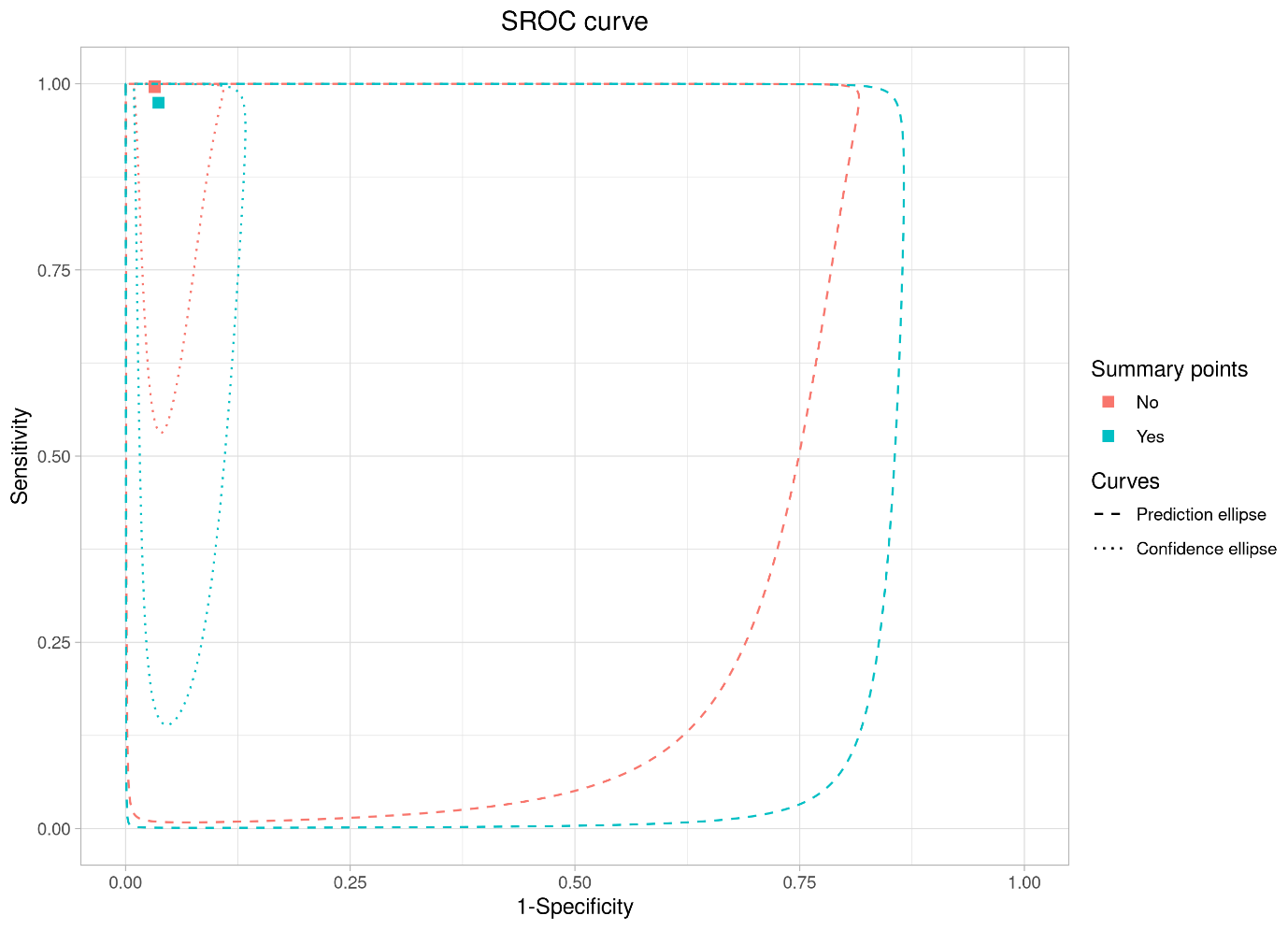


Supplementary Figure 16. Stacked bar chart of risk of bias domains, according to the Newcastle-Ottowa Scale.


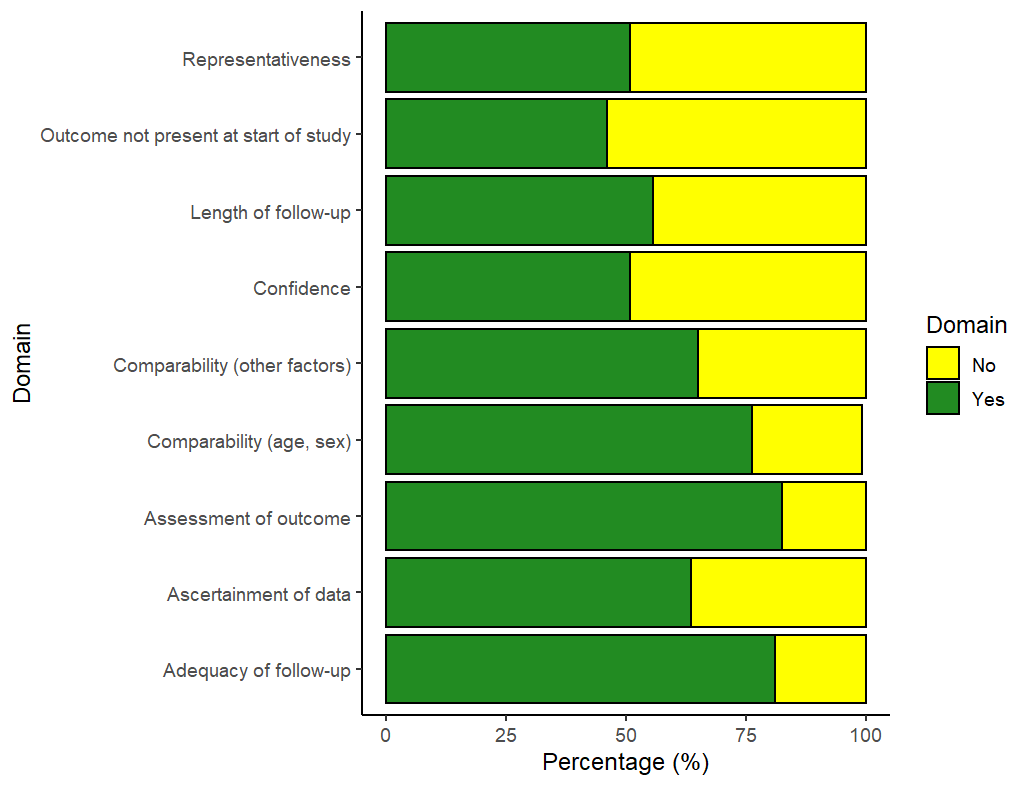


Supplementary Figure 17. Stacked bar chart of risk of bias domains, according to the Newcastle-Ottowa Scale for NICE included studies.


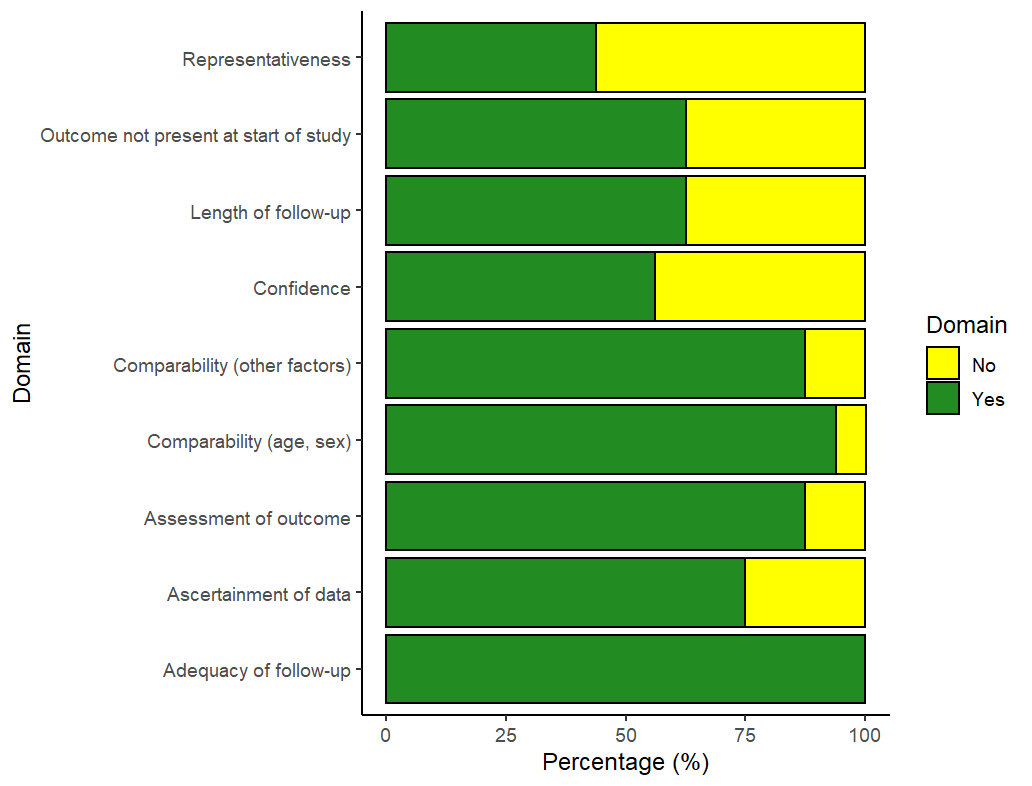


Supplementary Figure 18. Stacked bar chart of risk of bias domains, according to the Newcastle-Ottowa Scale for studies not included in NICE guidelines.


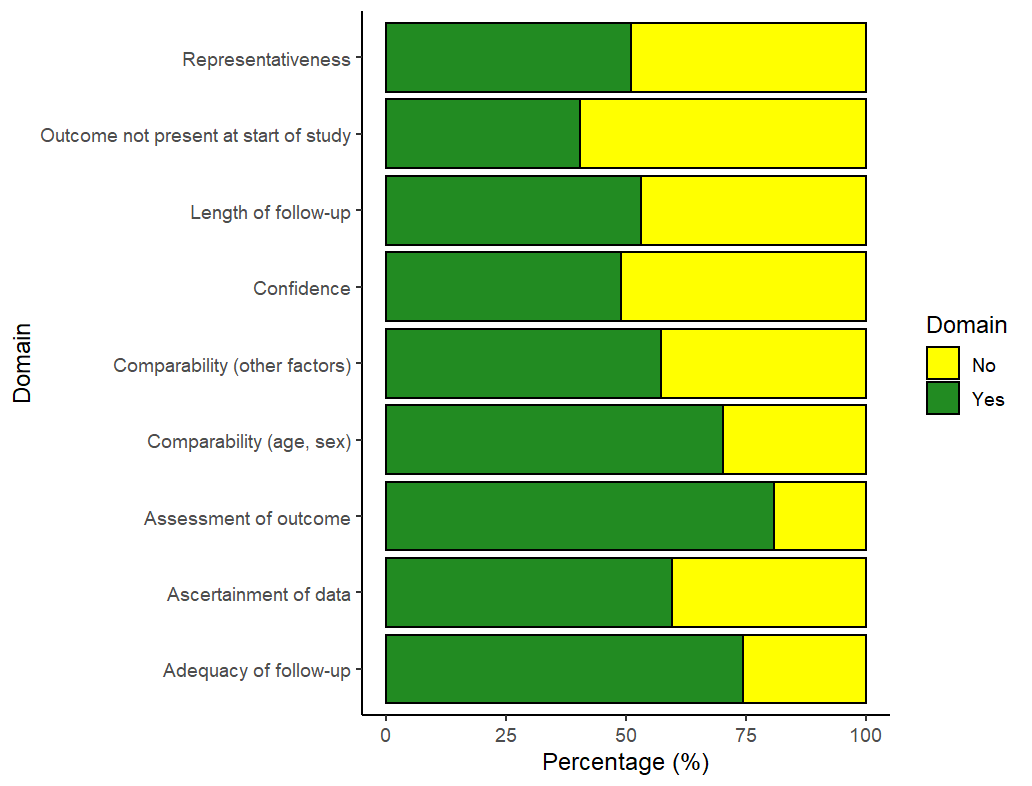

Supplement: Multimedia component 2 [file mmc2.docx]
